# Multifunctional Perovskite Photodetectors: From Molecular-Scale Crystal Structure Design to Micro/Nano-scale Morphology Manipulation

**DOI:** 10.1007/s40820-023-01161-y

**Published:** 2023-07-29

**Authors:** Yingjie Zhao, Xing Yin, Pengwei Li, Ziqiu Ren, Zhenkun Gu, Yiqiang Zhang, Yanlin Song

**Affiliations:** 1 College of Chemistry, Zhengzhou University, Zhengzhou, 450001 People’s Republic of China; 2 Henan Institute of Advanced Technology, Zhengzhou University, Zhengzhou, 450001 People’s Republic of China; 3 Key Laboratory of Green Printing, Institute of Chemistry, Chinese Academy of Sciences (ICCAS), Beijing, 100190 People’s Republic of China

**Keywords:** Perovskite materials, Crystal structure design, Micro/nano-structure manipulation, Working mechanism, Multifunctional photodetectors

## Abstract

Multifunctional photodetectors boost the development of traditional optical communication technology and emerging artificial intelligence fields, such as robotics and autonomous driving. However, the current implementation of multifunctional detectors is based on the physical combination of optical lenses, gratings, and multiple photodetectors, the large size and its complex structure hinder the miniaturization, lightweight, and integration of devices. In contrast, perovskite materials have achieved remarkable progress in the field of multifunctional photodetectors due to their diverse crystal structures, simple morphology manipulation, and excellent optoelectronic properties. In this review, we first overview the crystal structures and morphology manipulation techniques of perovskite materials and then summarize the working mechanism and performance parameters of multifunctional photodetectors. Furthermore, the fabrication strategies of multifunctional perovskite photodetectors and their advancements are highlighted, including polarized light detection, spectral detection, angle-sensing detection, and self-powered detection. Finally, the existing problems of multifunctional detectors and the perspectives of their future development are presented.

## Highlights

- Multidimensional detection of intensity, wavelength, polarization, and angle of the incidence light significantly accelerates the development of optical information technology and artificial intelligence fields.
- The first comprehensive overview of the advancement of multifunctional photodetectors for perovskite semiconductors ranging from polarized light detection, spectral detection, and angle-sensing detection to self-powered detection is summarized.
- The existing problems and perspectives are discussed which can inspire more researchers to rationally design new perovskite materials and micro/nano-structure for high-performance multifunctional photodetectors.

## Introduction

Photodetectors that convert optical information into electrical signals have become an indispensable part of modern optoelectronic devices and play an important role in traditional fields such as optical communication, imaging, sensing, and so forth [1–9]. In addition, with the rapid development of emerging technologies of artificial intelligence, such as robotics and autonomous driving, people have put forward higher requirements for the miniaturization, lightness, integration, and multifunctionality of photodetectors to detect more optical information such as intensity, spectrum, polarization, and incidence angle of light [10–12]. Although a large amount of research has been performed in the field of photodetectors and fruitful results have been achieved, the most attention has been paid to the detection of light intensity, and research on multifunctional photodetectors for the detection of polarization, spectrum, and incidence angle of light is still scarce [13–18]. Multifunctional photodetector means that in a single device, in addition to the detection of light intensity, it can also realize the detection of light polarization, spectrum, and incident angle information simultaneously [19–25]. The implementation of traditional multifunctional detectors relies on the physical combination of large optical lenses, gratings, waveplates, and multiple photodetectors, which results in large device size, complex structure, and high cost, and is not conducive to the integration of flexible multifunctional detectors in the future [21, 26, 27]. To further reduce the size and light weight of the devices, self-powered photodetectors also need to be developed [28].

Metal-halide perovskites are promising optoelectronic materials used in various devices such as solar cells, light-emitting diodes, lasers, memory, nonlinear optics, and photodetectors, which can be attributed to their diverse crystal structures, strong light absorption, tunable band gaps, high carrier mobility, solution processability and strong compatibility with flexible substrates [29–40]. Based on the outstanding material properties and diversity of perovskites, high-responsivity and low-detectivity perovskite photodetectors with broadband photodetection from the high-energy rays to the infrared region have been successfully fabricated [41–45]. Furthermore, multi-dimensional detection of optical information such as spectrum, polarization, and incident angle has also been achieved, providing new opportunities for photodetectors toward a new generation of low-cost optoelectronic device applications [21, 27, 46–48]. Despite the rapid progress in high-performance, multifunctional perovskite photodetectors, there are still remaining challenges, such as miniaturization, integration, and multifunctionality within a single device. Therefore, there is an urgent requirement to develop miniaturized and integrated multifunctional self-powered photodetectors to achieve simultaneously the detection of light intensity, polarization, spectrum, and incidence angle within a single device. In this respect, it is time to review perovskites and their multifunctional photodetector applications and provide promising strategies to promote the development of multifunctional photodetectors.

Herein, we systematically review the recent advances in perovskite multifunctional photodetectors. This review first briefly describes the fundamental properties and molecular-scale crystal structure design of perovskite materials and then analyzes the advantages and disadvantages of different processing techniques for micro/nano-scale morphology manipulation. After that, three operating mechanisms and important figures of merit of photodetectors are presented. Furthermore, we systematically described the development and analyzed the mechanisms of multifunctional perovskite photodetectors, mainly including polarized light detection, spectral detection, angle-sensing detection, and self-powered detection. Finally, we overview the remaining challenges and perspectives.

## Fundamental Principles

**Fig. 1:**
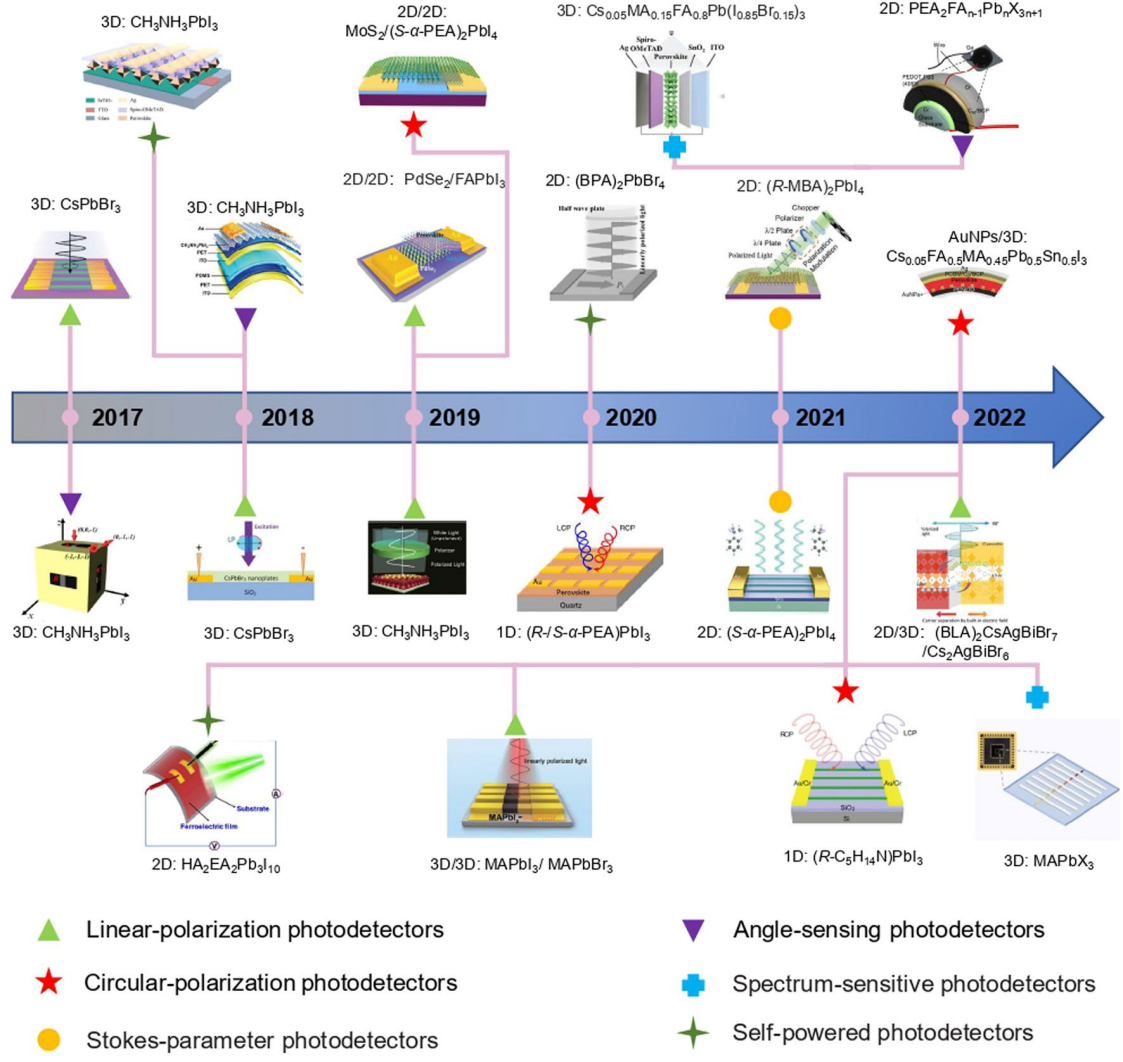
Overview of the development of multifunctional perovskite photodetectors in recent years, mainly including polarized light detection [21, 46, 47, 51, 55, 56, 58–64], spectral detection [27, 65], angle detection [54, 66, 67], and self-powered detection [68, 69]. Reproduced with permission from Ref. [21] Copyright 2019, Springer Nature; Reproduced with permission from Ref. [46] Copyright 2019, American Chemical Society; Reproduced with permission from Ref. [47] Copyright 2021, American Chemical Society; Reproduced with permission from Ref. [48] Copyright 2022, Wiley–VCH; Reproduced with permission from Ref. [49] Copyright 2018, The Royal Society of Chemistry; Reproduced with permission from Ref. [50] Copyright 2019, Springer Nature; Reproduced with permission from Ref. [51] Copyright 2022, Wiley–VCH; Reproduced with permission from Ref. [55] Copyright 2017, Wiley–VCH; Reproduced with permission from Ref. [56] Copyright 2020, Wiley–VCH; Reproduced with permission from Ref. [58] Copyright 2022, Elsevier; Reproduced with permission from Ref. [59] Copyright 2018, Wiley–VCH; Reproduced with permission from Ref. [60] Copyright 2019, Wiley–VCH; Reproduced with permission from Ref. [61] Copyright 2019, Wiley–VCH; Reproduced with permission from Ref. [62] Copyright 2021, Wiley–VCH; Reproduced with permission from Ref. [63] Copyright 2022, Wiley–VCH; Reproduced with permission from Ref. [64] Copyright 2022, Wiley–VCH; Reproduced with permission from Ref. [27] Copyright 2022, Wiley–VCH; Reproduced with permission from Ref. [65] Copyright 2021, Wiley–VCH; Reproduced with permission from Ref. [54] Copyright 2017, American Chemical Society; Reproduced with permission from Ref. [66] Copyright 2018, Wiley–VCH; Reproduced with permission from Ref. [67] Copyright 2022, Springer Nature; Reproduced with permission from Ref. [68] Copyright 2018, American Chemical Society; Reproduced with permission from Ref. [69] Copyright 2022, American Chemical Society

Due to the excellent optoelectronic properties of perovskite materials, it is widely used in a variety of high-performance photodetectors with wavelengths ranging from X-ray to near-infrared regions [49–51]. In recent years, with the increased crystal structure design of perovskite materials and the rapid advancement of perovskite processing technology for morphology manipulation, the research of multifunctional photodetectors has also achieved rapid progress [52, 53]. Besides light intensity detection, the detection of optical information such as spectrum, polarization, and incident angle has also been realized (Fig. 1) [47, 48, 54–58]. In this section, we will focus on an overview of the molecular-scale crystal structure design of perovskite materials and micro/nano-scale morphology manipulation by different processing techniques, the operating mechanisms of photodetector devices, and figures of merit of photodetectors.

### Molecular-Scale Crystal Structure Design of Metal Halide Perovskites

**Fig. 2:**
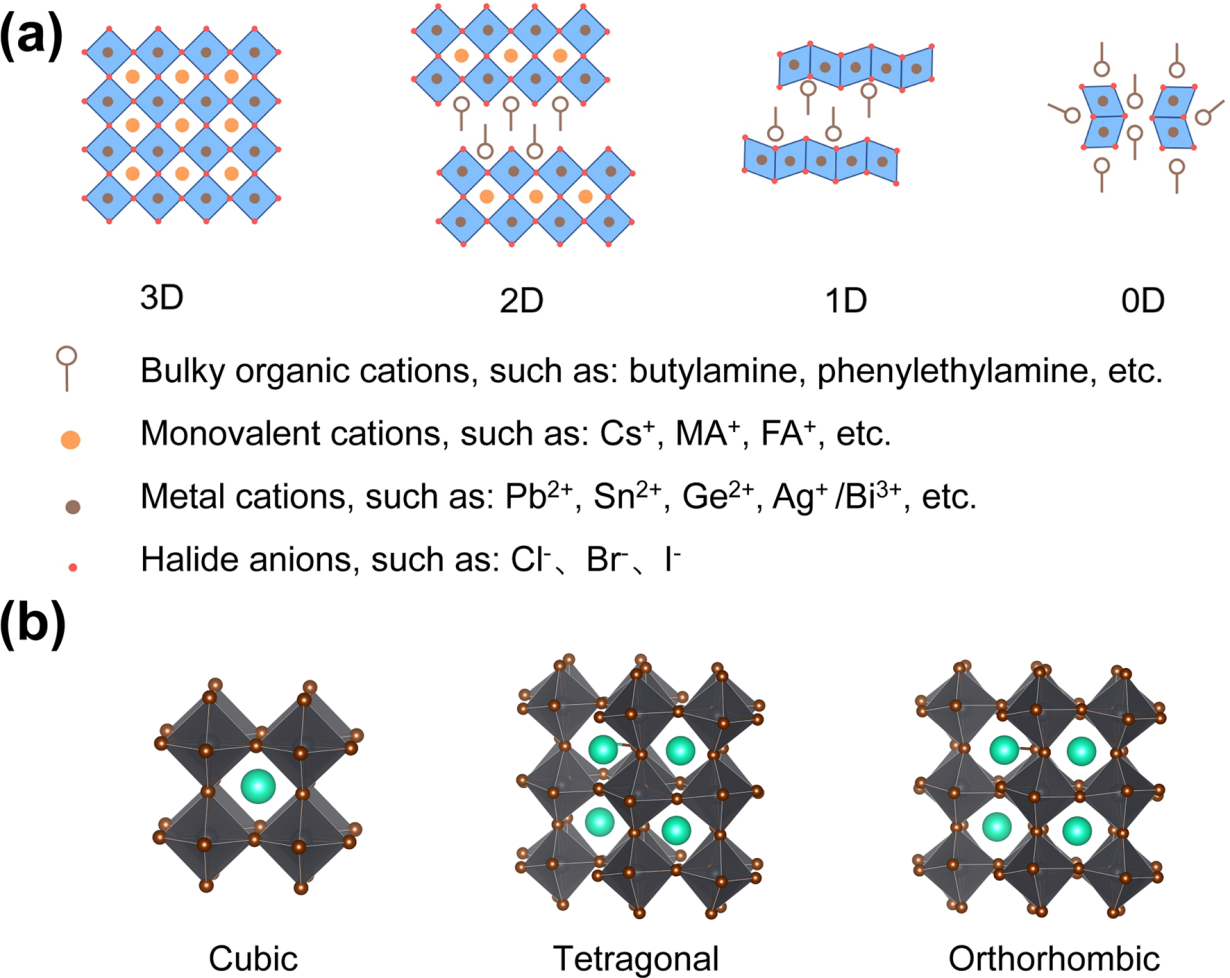
Classification of perovskite materials. **a** Diversity of perovskite materials, mainly including 3D, 2D, 1D, and 0D structures. **b** Crystal structures of perovskite materials

Perovskite materials generally refer to materials with a perovskite crystal structure, which can be denoted generally as ABX_3_, where A and B are cations and X is the anion [5]. B and X form the octahedral framework of metal halide perovskite, which can be expressed as [BX_6_]^4−^. Based on their octahedral connections, perovskites can be classified into three-dimensional (3D), two-dimensional (2D), one-dimensional (1D), and zero-dimensional (0D) structures [5], as shown in Fig. 2a. The 3D perovskite network is constructed by connecting octahedra with shared angles of [BX_6_]^4−^. The composition of 3D perovskite can be tuned by selecting different A-site cations, B-site cations, and X-site anions. It is also possible to control the crystal growth kinetics of 3D perovskites, resulting in the formation of low-dimensional nanostructures such as quantum dots, nanowires, and nanosheets [70]. Low-dimensional perovskite is produced by reducing the connectivity of [BX_6_]^4−^. 2D perovskite is formed by the insertion of larger organic cation layers into the octahedral network of 3D perovskite, which can be generally expressed as (LA)_*m*_(A)_*n*-1_B_*n*_X_3*n*+1_, where LA is the larger organic cation and *n* is the layer number of the 2D perovskites [71]. In 1D perovskite, the highly distorted coplanar octahedra [BX_6_]^4−^ are surrounded by larger organic cations [58]. Further reduction connectivity of octahedral [BX_6_]^4−^ network yields 0D perovskite [72]. In addition to the dimensional classification of perovskite materials, the crystal structures of perovskite can also be transformed by the introduction of different interlayer molecules and inorganic ions [73]. A large number of symmetric and asymmetric perovskite crystal materials are synthesized through the design of molecular structures and inorganic ions. The common crystal structures in 3D perovskite are the cubic phase, orthorhombic phase, and tetragonal phase (Fig. 2b). The introduction of large intercalating amine molecules further enriches the crystal structure of perovskite materials, yielding monoclinic, triclinic, and other crystal structures. In particular, the introduction of chiral intercalating molecules generates the structure of the chiral space group [21]. In these structures, asymmetrical crystal structures consisting of orthorhombic, monoclinic, and triclinic are commonly demonstrated in perovskites, empowering them with intrinsic anisotropic properties for highly polarization-sensitive photodetectors.

### Micro/Nano-scale Morphology Manipulation

The functionality of perovskite devices depends not only on the properties of the material itself but is also closely linked to its nanometer-scale morphology. Multi-scale morphology engineering allows the integration of novel properties into a single material, which facilitates the development of ultra-compact multifunctional devices [47]. Perovskite morphology manipulation depends on the development of processing technology. Traditionally, the preparation of perovskite optoelectronic devices is mainly based on spin-coating and blade-coating methods [31]. For the formation of thin films, precise control of perovskite crystallization can effectively optimize the film morphology including grain size, surface coverage, and thickness, which determine the properties of light absorption and charge transport within perovskite films. To date, perovskite polycrystalline films have been intensively studied and photodetectors have been developed [8]. However, a large number of defects within polycrystalline films limit the device's performance. In addition, a large amount of waste of materials and the non-tunable nano-scale morphology hinders its miniaturization, integration, and multi-functionalization.

**Fig. 3:**
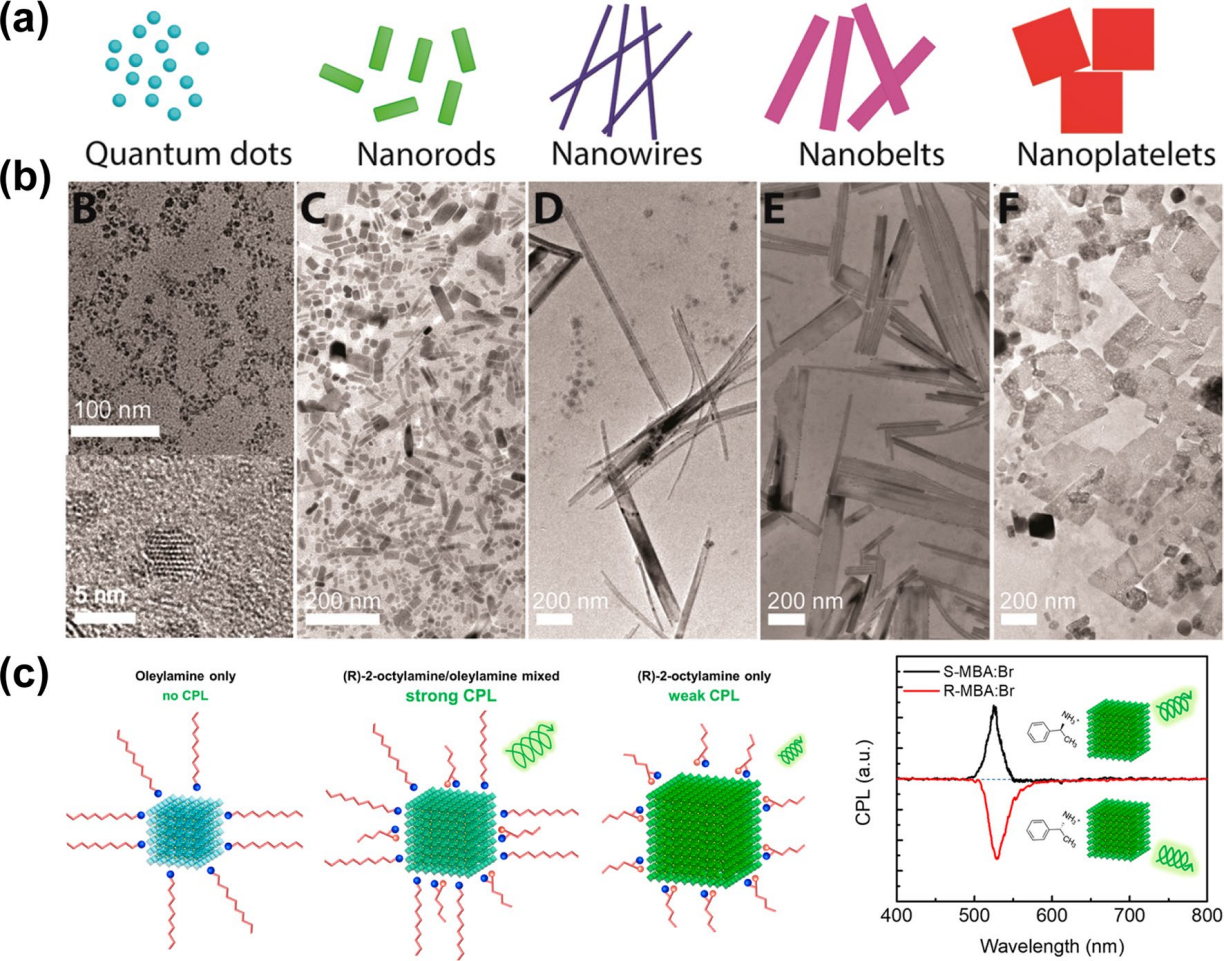
Morphology management of perovskite nanocrystals using hot-injection process. **a** Schematic diagram and **b** TEM images of perovskite nanocrystals with different shapes. Reproduced with permission from Ref. [74] Copyright 2019, American Chemical Society. **c** Schematic diagram and CPL of ligand-treated FAPbBr_3_ quantum dots. Reproduced with permission from Ref. [76] Copyright 2020, American Chemical Society

To achieve the control of perovskite morphology, the direct method is chemical thermal injection synthesis [74]. A series of perovskite nanocrystals with different morphologies, such as nanorods, nanowires, nanobelts, and nanoplatelets have been synthesized (Fig. 3a, b) [74]. Numerous new optoelectronic functions have been introduced by the change of perovskite nanocrystal morphology and their assembly shape. For example, Yang et al. achieved high linear-polarization emission by synthesizing perovskite nanowires with a large aspect ratio and realized the linear-polarization imaging function [75]. Circularly polarized light emission from perovskite quantum dots was realized by introducing chiral surface ligands (Fig. 3c) [76]. Although the direct chemical synthesis method can control the morphology of perovskite at the nanoscale, its overall performance needs to be further improved. The main reasons limiting its optoelectronic performance can be attributed to the following three aspects. First, a large number of long-chain organic ligands exist on the surface of nanocrystals, which are not conducive to carrier transport in optoelectronic devices; Second, a large number of defects usually appear on the surface of nanocrystals due to the rapid nucleation growth process as well as the large specific surface area; Third, process of film, resulting in limited carrier transport.

**Fig. 4:**
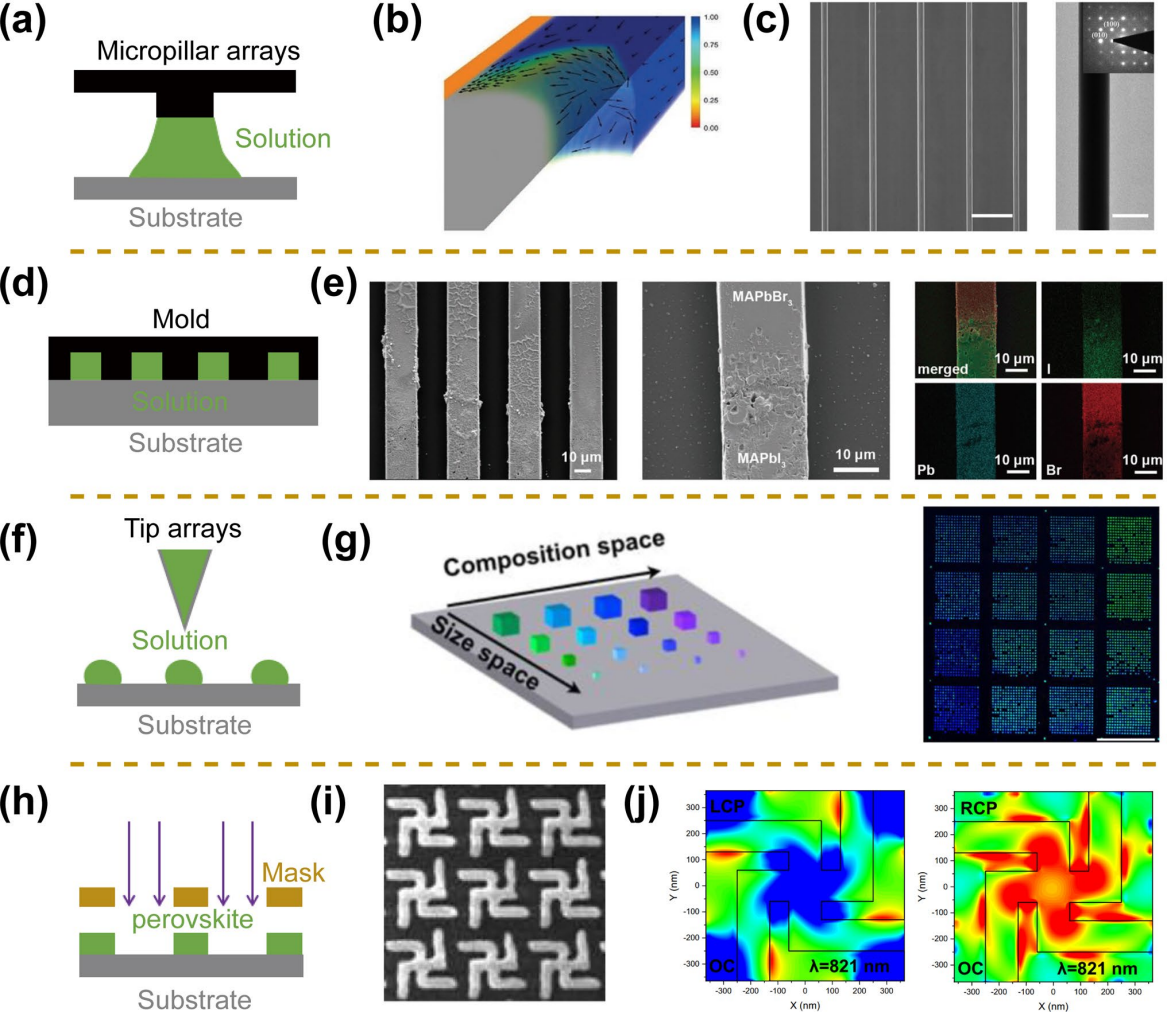
Morphology management of perovskites using different processing technology. **a** Schematic diagram of the capillary liquid bridge method. **b** Fluid simulation diagram of the capillary liquid bridge. **c** SEM and TEM images of perovskite nanowire arrays. Reproduced with permission from Ref. [55] Copyright 2017, Wiley–VCH. **d** Schematic diagram of PDMS template method. **e** SEM and EDS mapping images of perovskite heterojunction arrays. Reproduced with permission from Ref. [63] Copyright 2022, Wiley–VCH. **f** Schematic diagram of dip pen printing method. **g** Confocal PL mapping of 16 individual perovskite arrays. Reproduced with permission from Ref. [77] Copyright 2022, American Chemical Society. **h** Schematic diagram of etching technique. **i** SEM image of perovskite chiral metasurfaces. **j** Color maps showing dissimilar spatial distributions of optical chirality for a left-handed chiral gammadion meta molecule at different circular polarization illumination. Reproduced with permission from Ref. [78] Copyright 2022, Springer Nature

In addition to the thermal injection method, to accelerate the development of integrated, miniaturized, and multifunctional perovskite photodetectors, a series of processing methods for micro/nano-scale perovskite arrays have also been rapidly developed [53, 74]. Solution-processed assembly technologies, mainly including nanoimprinting, dip-pen printing, inkjet printing, and capillary-bridge confined assembly methods, have been used for the preparation of large-area micro/nanostructure. The advantage of these technologies includes two aspects. First, the confinement or partitioning of the macroscopic liquid is realized. Second, micro/nanostructure arrays with controllable positions can be fabricated [75]. For example, Wu et al. reported the fabrication of perovskite nanowire arrays based on the capillary liquid bridge-induced assembly method, which exhibited excellent linearly polarized light absorption properties arising from large aspect ratios and achieved linearly polarized light detection (Fig. 4a–c) [55]. The long-range ordered assembly of perovskite nanowire arrays can be attributed to the directional mass transfer process at the capillary trailing. Sun et al. achieved the fabrication of perovskite heterojunction arrays using the PDMS template-induced assembly method and finally realized the high-performance linearly polarized light detection (Fig. 4d, e) [63]. In addition, Mirkin et al. achieved the preparation of large-area gradient bandgap perovskite nanoarrays based on the dip-pen printing method (Fig. 4f, g) [77]. The etching technique is also widely used for the nanoarray processing of perovskite. Cesare Soci et al. fabricated nanoscale perovskite metasurfaces based on a focused ion beam etching process that enables circularly polarized light absorption (Fig. 4h–j) [78]. However, its high preparation cost and damaging effects on perovskite materials restrict its widespread application [52, 53]. Based on these technologies, multifunctional perovskite photodetectors with different morphology have been developed, mainly including polarized light detection, spectral detection, angle-sensing detection, and self-powered detection.

### Types of Photodetectors

**Fig. 5:**
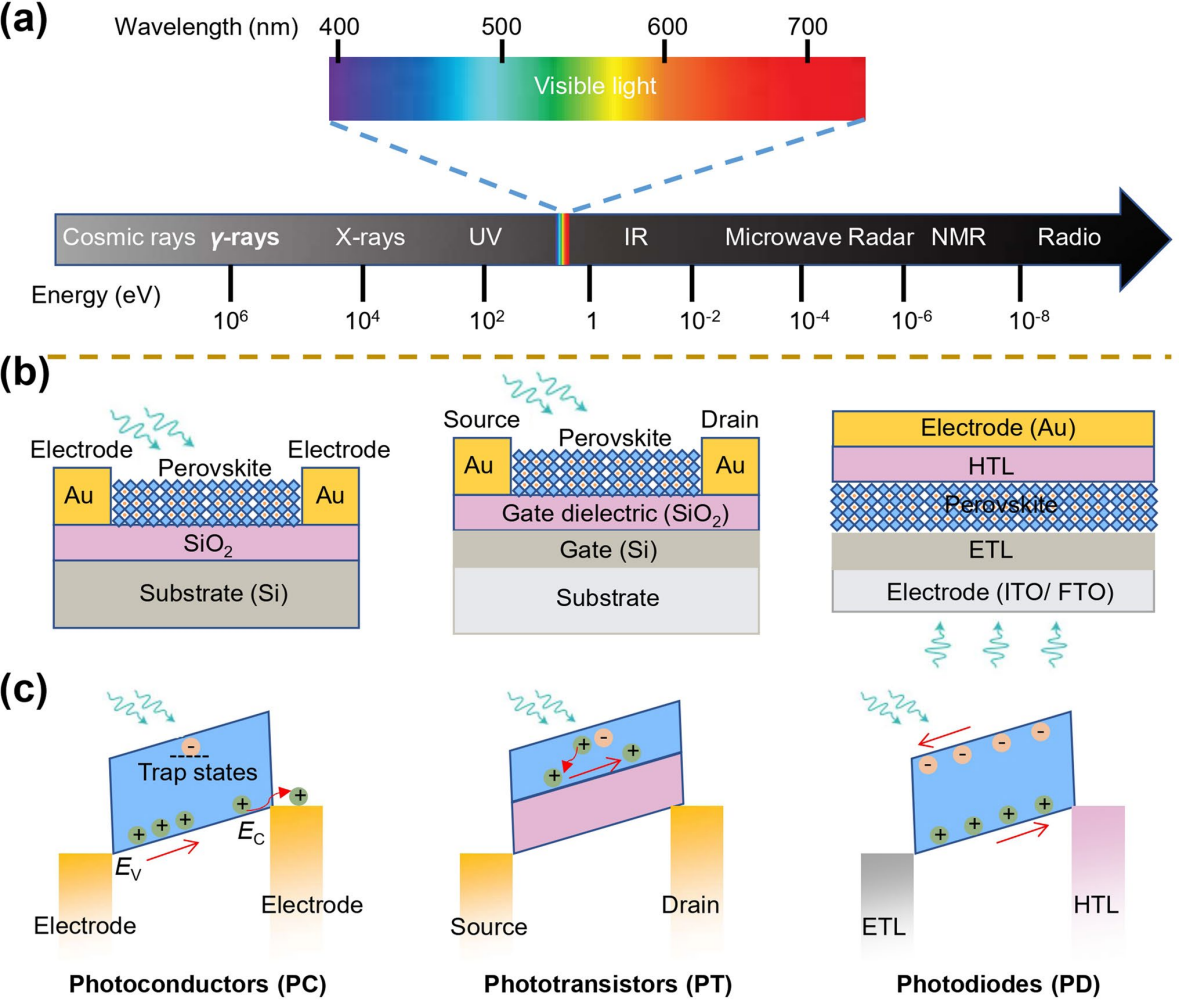
Classification of perovskite photodetectors. **a** The detection wavelength of photodetectors. **b** Device structures and **c** their operating mechanisms of perovskite photodetectors

The diversity of perovskite materials and processing technologies yield diverse perovskite photodetectors. According to the detection wavelength, perovskite photodetectors can be divided into X-ray detectors, ultraviolet detectors, visible light detectors, and near-infrared detectors, which can be widely used in medical imaging, sensing, and night vision, etc. (Fig. 5a, b) [45, 51, 79]. Based on the different photodetector structures and operating mechanisms, perovskite photodetectors can be classified into three device types: photoconductors, photodiodes, and phototransistors [9, 80]. The detailed device structure and its operating mechanism are described below (Fig. 5b, c). A photoconductor is a planar device with a two-terminal structure. The photoactive layer acts as a channel between the electrodes enabling the detection mechanism relatively simple. In a photoconductor, light-generated carriers are separated by an externally applied bias voltage and then collected at each electrode. In this device structure, one type of carrier is typically trapped in the trap center with a longer lifetime. Meanwhile, another type of carrier can be recirculated through an external circuit until it recombines with the opposite carrier, thus yielding a photoconductive gain. This gain mechanism leads to remarkably high external quantum efficiency and responsivity compared to photodiodes. To reduce the noise current, the phototransistor has an additional gate electrode and a dielectric layer in the device structure. By changing the value of the gate voltage, the phototransistor can modulate the semiconductor channel, thus regulating the channel conductivity and suppressing the dark current. In this device structure, the device has a large photoconductive gain and low noise and thus has a high sensitivity. Photodiodes adopt a vertical device structure similar to photovoltaic devices, where the electrons and holes generated by light are separated by a built-in potential formed by a p–n junction and then moved to opposite electrodes through an electron/hole transport layer. In general, different device structures lead to significant differences in the performance of photodetectors. According to the spatial layout of the photoactive layer and electrodes, vertical devices with smaller electrode spacing and shorter carrier transfer distance possess the characteristics of fast response speed and low driving voltage. In contrast, planar devices present the characteristics of slow response speed and high driving voltage due to large electrode spacing, resulting in lower detectivity and a narrower linear dynamic range [5].

### Figure of Merits for Photodetectors

To better characterize the performance of the photodetectors, the figure of merits for photodetectors is presented. In addition, the performance metrics of the multifunctional photodetectors are also summarized below.

1. On/off ratio
  The on/off ratio refers to the ratio of photocurrent (*I*_photo_) and dark current (*I*_dark_) under specific light wavelength, light intensity, and bias voltage, which is mainly dependent on the photoelectric conversion and charge transport ability of the perovskite active layer. To increase the on/off ratio, the photocurrent value should be increased and the dark current value should be suppressed.
2. Responsivity (*R*)
  *R* is a metric that quantifies the ability of a photodetector converting light into current. *R* is the ratio of photocurrent and incident light power (*P*), and its value depends on the wavelength of the incident light. Responsivity can be expressed as, *R*(AW^−1^) = (*I*_photo_−*I*_dark_)/*PA*, Where *A* is the effective area of the device.
3. External quantum efficiency (*EQE*)
  The external quantum efficiency refers to the ratio of the number of carriers collected in the device and the number of incident photons. It can be expressed as, *EQE* = *Rhc*/*eλ*, Where *h, c, e, λ* are Planck constant, speed of light, electron charge, and wavelength of light, respectively.
4. Response time
  Response time usually refers to the response speed of the device and can be divided into rise time (*τ*_rise_) and fall time (*τ*_fall_). *τ*_rise_ can be defined as the time in which the photocurrent rises from 10 to 90% of the maximum current value, whereas *τ*_fall_ refers to the time that the photocurrent falls from 90 to 10% of the maximum current value.
5. Detectivity (D*)
  The specific detectivity represents the sensitivity of the device and is used to evaluate the ability of the device for detecting the weakest light, which is dependent on the responsivity and noise current (*i*_noise_) of the photodetector. The unit of specific detectivity is Jones (cm Hz^1/2^ W^−1^). It can be expressed as, D*(Jones) = *R*(*AB*)^1/2^/*i*_noise_ = (*AB*)^1/2^/*NEP*, Where *A, B, NEP* are the active area, bandwidth, and noise equivalent power of the device, respectively.
6. Linear-polarization anisotropy ratio (*g*)
  To quantify the linear polarization light detection ability of the photodetector, the linear-polarization anisotropy ratio is introduced. It refers to the ratio of the maximum photocurrent (*I*_max_) and the minimum photocurrent (*I*_min_). It can be given as, *g* = *I*_max_/*I*_min_.
7. Circular-polarization anisotropy factor (*g*_res_)
  To quantify the circular-polarization light detection ability of photodetectors, the circular-polarization anisotropy factor of responsivity is introduced. It represents the difference in the absorption ability of the same perovskite material for left- and right-handed circularly polarized light. It can be expressed as,* g*_res_ = 2(*R*_L_−*R*_R_)*/*(*R*_L_ + *R*_R_), Where *R*_L_ and* R*_R_ are the responsivity under left- and right-handed circularly polarized light illumination, respectively.
8. Spectral response range
  For perovskite photodetectors, the different response wavelengths boost the different applications of devices. In particular, the wide spectral response facilitates the full-spectrum detection of perovskite spectrometers. The energy of the response wavelength of the device is usually larger than the band gap of the perovskite material, and its spectral response range can be defined as the wavelength that the detector can detect.
9. Spectral resolution
  For perovskite spectrometers, miniaturized and integrated spectrometers with high spectral resolution are necessary because of their wide application in the optoelectronics fields. The spectral resolution can be defined as the minimum full width at half maximum of the reconstructed spectrum for perovskite spectrometers.
10. Response range of incidence angle
  For angle-sensing photodetectors, a wide range of incident angle response/recognition presents a great advantage in intraoperative navigated surgery, artificial intelligence, and autonomous driving. The range of incident angle response can be defined as the maximum angle that the device can detect/recognize.

## Multifunctional Perovskite Photodetectors

The detection of optical information mainly includes the intensity, polarization, spectrum, and incidence angle of light. With the emergence of new technologies, photodetectors will continuously develop in the direction of miniaturization, integration, and multi-functionalization. Based on the excellent optoelectronic properties, diverse crystal structure, and morphology manipulation of perovskite materials, a variety of multifunctional photodetectors with high responsivity and detectivity have been developed [27, 47, 48, 54, 69]. The multifunctional photodetector has numerous promising applications. For example, polarization photodetectors can be used for imaging, environmental monitoring, quantum communication, etc. [47, 55]; spectrum-sensitive /angle-sensitive photodetectors can be used for autonomous driving, robot vision, etc. [19, 23, 24, 26, 54]. Anisotropic perovskite with low-symmetry crystal structure possesses the property of linearly polarized light detection [81]; the separation of positive and negative charges of ferroelectric perovskite makes them self-powered detection [69]; the introduction of chiral amines yields chiral perovskite with the property of circularly polarized light detection [21]. Apart from utilizing the properties of the perovskite crystal structure itself, the micro- and nanostructure morphology can further extend the functionalization of photodetectors [55, 61]. The perovskite nanowire arrays are prepared based on nanoimprinting/capillary-bridge confined assembly method, and the large aspect ratio endows them with linearly polarized light detection properties [55, 61]; the metasurface prepared based on the etching method endows the perovskite with circularly polarized light detection properties [57].

### Linear-Polarization Photodetectors

In addition to light intensity detection, polarization-sensitive photodetectors have also attracted a lot of research attention and have important applications in polarization imaging, high contrast polarization mirrors, sensing, optical radar, environmental monitoring, and other fields [47, 55, 82, 83]. Unlike traditional detection techniques, polarization detection can not only distinguish the signal intensity from the target and the background but also accurately recognize the polarization information of the target [83]. Therefore, it can efficiently suppress background interference signals and promote the progress of its detection and imaging. When light interacts with matter, the influence of the electric vector is much greater than that of the magnetic field vector, and the direction of vibration of the electric vector is always perpendicular to the propagation direction of light. Among them, linearly polarized light refers to the trajectory of its electric vector as a straight line during the propagation of light and has a fixed vibrational plane. At present, the implementation of linearly polarized light detection is mainly based on the following mechanisms: (i) intrinsic anisotropic crystal structures; (ii) perovskite heterojunction structure; and (iii) 1D geometry morphology. The performance of representative linear-polarization photodetectors is summarized in Table 1.

**Table 1:** A summary of important figures of merit of representative perovskite linear-polarization photodetectors

Device structure	Materials	Polarization mechanism	*R* (A W^−1^)	D* (Jones)	Wavelength (nm)	Polarization ratio (*g*)	References
Photoconductor	(BA)_2_(MA)Pb_2_Br_7_ crystal	Anisotropic crystal structures	–	10^9^	405	1.9	[81]
Photoconductor	(FPEA)_2_PbI_4_ crystal	Anisotropic crystal structures	3.2	3.8 × 10^11^	520	2.1	[84]
Photodiode	PEA_2_MA_4_(Sn_0.5_Pb_0.5_)_5_I_16_ film	Anisotropic crystal structures	0.35	1.53 × 10^12^	900	2.4	[85]
Photoconductor	PdSe_2_/FAPbI_3_ heterostructure	Heterogeneous structure	0.313	10^13^	800	6.04	[60]
Photoconductor	(BLA)_2_CsAgBiBr_7_/Cs_2_AgBiBr_6_ heterocrystals	Heterogeneous structure	0.6	3.510^14^	405	9	[64]
Photoconductor	MAPbI_3_–MAPbBr_3_ microwire heterojunctions	Heterogeneous structure	1207	2.8 × 10^13^	650	8.2	[63]
Photoconductor	CH_3_NH_3_PbI_3_ nanowire	Geometry morphology	4.95	2 × 10^13^	530	2.6	[86]
Photoconductor	CsPbBr_3_ nanowire arrays	Geometry morphology	1000	–	470	2.6	[55]
Photoconductor	CH_3_NH_3_PbI_3_ microwire	Geometry morphology	7	–	405	1.57	[87]
Photoconductor	MAPbBr_3_ crystal	Geometry morphology	1026.5	1.9 × 10^13^	650	9.1	[88]

#### Intrinsic Anisotropic Crystal Structures

**Fig. 6:**
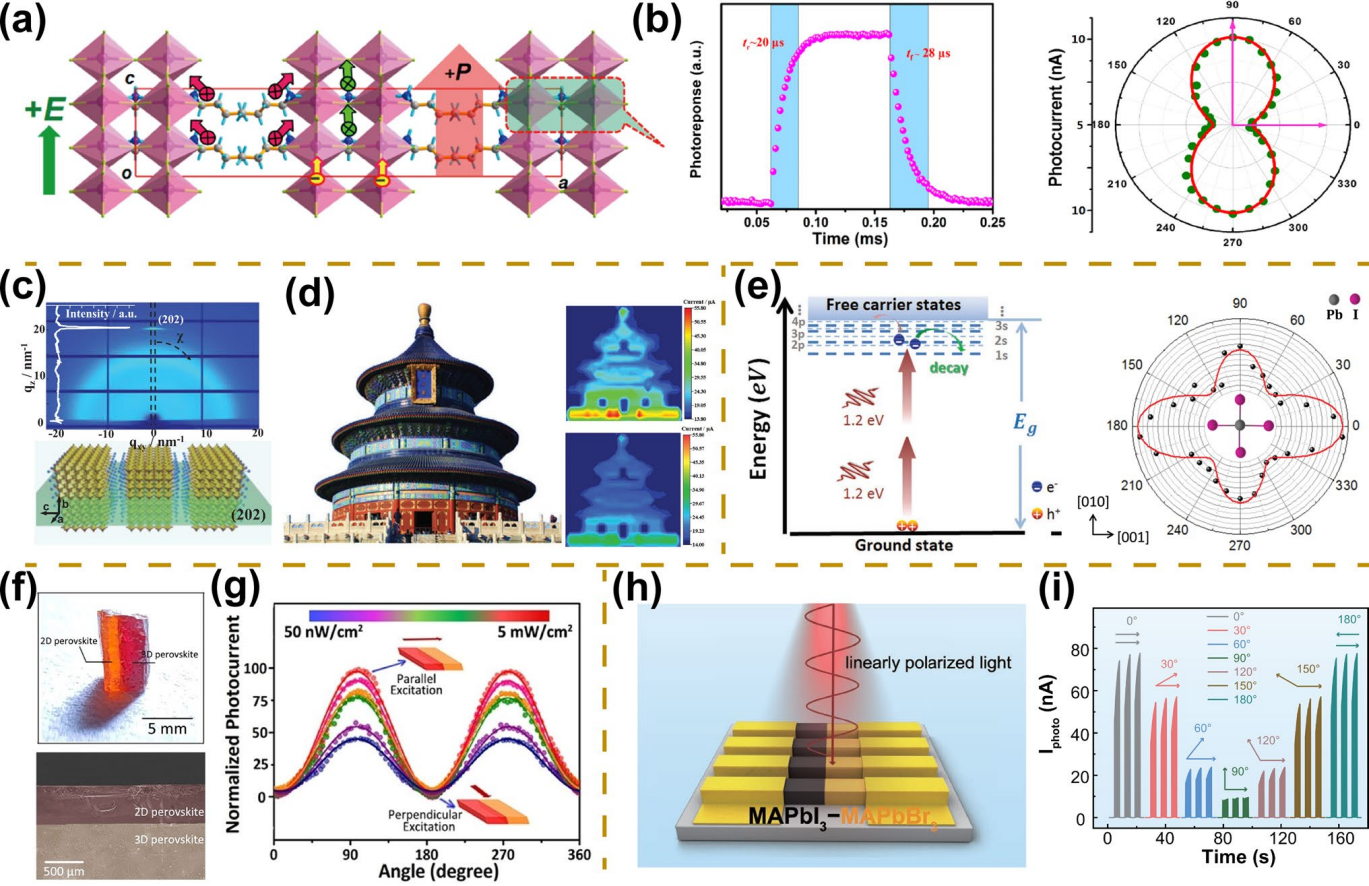
Linearly polarized light detection. **a** Schematic diagram of layered perovskite crystals, (BA)_2_(MA)Pb_2_Br_7_. The red arrow represents the possible direction of electric polarization. **b** Angle-dependent photocurrent under different polarized light irradiation (left). Time-dependent photoresponse (right). Reproduced with permission from Ref. [81] Copyright 2019, American Chemical Society. **c** Schematic diagram of (202) crystallographically oriented 2D perovskite polycrystalline films, PEA_2_MA_4_(Sn_0.5_Pb_0.5_)_5_I_16_. **d** Polarized light imaging with the 0° polarization light (top) and 90° polarization light (bottom). Reproduced with permission from Ref. [85] Copyright 2021, Wiley–VCH. **e** Energy level diagram of two-photon absorption (left) and angle-dependent photocurrent under different polarized light irradiation (right). Reproduced with permission from Ref. [89] Copyright 2019, Wiley–VCH. **f** The optical photo (top) and cross-section image (bottom) of 2D/3D double perovskite heterojunctions. **g** Angle-dependent photocurrent under different polarized light irradiation. Reproduced with permission from Ref. [64] Copyright 2022, Wiley–VCH. **h** Schematic diagram of polarized photodetector based on MAPbBr_3_-MAPbI_3_ lateral heterojunctions. **i** Angle-dependent photocurrent under different polarized light irradiation. Reproduced with permission from Ref. [63] Copyright 2022, Wiley–VCH

The intrinsic crystal anisotropy of perovskite materials provides an intriguing opportunity for linearly polarized photodetectors [82]. Sun et al. reported a novel two-dimensional ferroelectric perovskite that exhibits large spontaneous polarization of 3.6 μC cm^−2^ (Fig. 6a). The unique anisotropic structure enables the perovskite to exhibit large anisotropic optical absorption in the parallel polarization direction (a stronger optical absorption) and the perpendicular polarization direction (a weaker optical absorption). Based on this ferroelectric perovskite crystal, a photodetector with a large shortwave *g* of 2 is eventually obtained (Fig. 6b). Also, the large detectivity up to 10^9^ Jones as well as the fast response speed of 20 μs are demonstrated for the perovskite photodetector, which can be attributed to the large spontaneous polarization favoring the dissociation of photo-induced carriers [81].

Although the above photodetectors enable linearly polarized light detection, their narrow spectral response range mainly locates in the UV region, thus limiting their application toward full-spectrum detection. To achieve broad-spectrum detection, Zeng et al. reported pure (202) crystallographic orientation of solution-processed 2D perovskite polycrystalline films, PEA_2_MA_4_(Sn_0.5_Pb_0.5_)_5_I_16_ (PEA = phenylethylammonium, MA = methylammonium), exhibiting anisotropic optoelectronic properties, realizing broad-spectrum polarization detection from 300 to 1050 nm (Fig. 6c) [85]. The pure crystallographic orientation can be attributed to the addition of NH_4_SCN and NH_4_Cl, which regulates the nucleation crystallization of perovskite films. Based on purely oriented anisotropic polycrystalline films, a high-performance polarized photodetector is demonstrated with a *g* of 2.4 at 900 nm, a response bandwidth of 900 kHz, an *I*_on_/*I*_off_ ratio of 3 × 10^8^, and a specific detectivity of 1.53 × 10^12^ Jones. In addition, high-resolution polarization imaging is confirmed to meet the needs of practical applications (Fig. 6d). Nevertheless, the detection of polarized light at wavelengths above 1000 nm is challenging due to the limited bandgap of perovskite materials. In this regard, Ji et al. fabricated high-performance sub-bandgap photodetectors based 2D (C_4_H_9_NH_3_)_2_(CH_3_NH_3_)_*n*−1_Pb_*n*_I_3*n*+1_ perovskites, which present excellent detection performance in the near-infrared region [89]. Sub-bandgap detectors also exhibit a sensitive response to polarization light with a considerable anisotropy in their degenerate two-photon absorption (D-2PA) coefficients (*β*_[001]_/*β*_[011]_) of 2.4 (Fig. 6e). Finally, it is demonstrated that sub-bandgap photodetectors also present great potential for three-photon absorption photodetectors, which will facilitate the development of infrared polarization photodetectors.

#### Perovskite Heterogeneous Structure

Although 2D perovskite crystals with intrinsic crystal anisotropy have been used as active layers for linearly polarized photodetectors, the polarization anisotropy ratio of these devices is generally less than 3, thus limiting their application as polarized photodetectors. Heterostructures composed of several optoelectronic materials with different physical properties are the basis for the integration of multifunctional high-performance micro-/nanostructure devices [90–94]. Compared with single-component systems, heterostructures can effectively manipulate device performance, integrate multiple functions, and generate specific applications. Recent works have shown that the construction of heterostructures can effectively enhance the polarization sensitivity of perovskite photodetectors, which can be attributed to the fact that the separation of electrons and holes following the direction of the "built-in electric field" is amplified under linearly polarized light excitation [63, 64].

Based on 2D/3D ((BLA)_2_CsAgBiBr_7_/Cs_2_AgBiBr_6_) double perovskite heterojunctions, Luo et al. first demonstrated a linearly polarized light detection with a large photocurrent parallel to the direction of the built-in electric field and smaller current perpendicular to the direction of the built-in electric field, yielding large *g* up to 9, which can be attributed to the combination of well-defined interface and built-in electric field provided by heterogeneous integration (Fig. 6f, g) [64]. Moreover, the heterojunction photodetector enables the detection of very weakly polarized light with a minimum intensity of 50 nW cm^2^. Compared to the above bulky single-crystal devices, microwire array devices present more advantages of miniaturization and integration. Sun et al. [63] fabricated high-quality MAPbBr_3_–MAPbI_3_ perovskite lateral heterojunctions using a two-step nanoimprinting method (Fig. 6h). Benefiting from strict arrays, high crystalline quality, and sharp heterogeneous interfaces, high-performance polarized photodetectors are successfully prepared with a responsivity of 1207 A W^−1^, detectivity of 2.78 × 10^13^ Jones, and ultra-high polarization ratio of 8.2 (Fig. 6i). Due to the outstanding mechanical properties of the microwire array, the detector also exhibits excellent flexibility. In addition, the heterojunction construction of two-dimensional materials (such as PdSe_2_ and hBN) with perovskite also shows great potential for applications in the field of polarization photodetection [60].

#### 1D Micro/Nano-scale Geometry Morphology

**Fig. 7:**
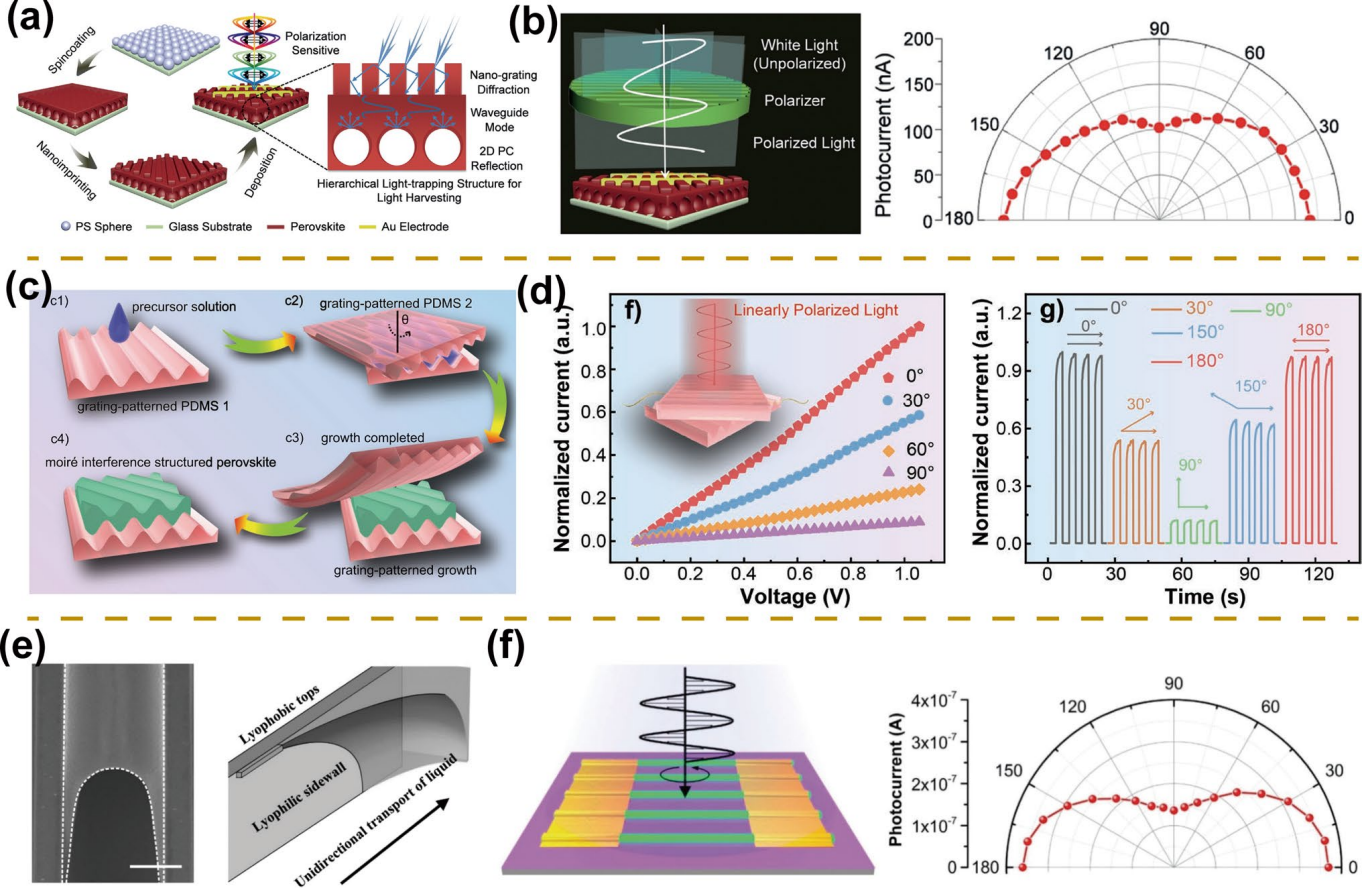
Linearly polarized light detection. **a** Schematic diagram of the preparation process for 1D nano-grating structure atop a two-dimensional photonic crystal structure. **b** Schematic diagram of polarized light detector (left). Angle-dependent photocurrent under different polarized light irradiation (right). Reproduced with permission from Ref. [61] Copyright 2019, Wiley–VCH. **c** Schematic diagram of in-situ encapsulated Moiré lattice-perovskite films. **d** Angle-dependent *I*-*V* curves (left) and *I*-*t* curves (right) under different polarized light irradiation. Reproduced with permission from Ref. [88] Copyright 2022, Wiley–VCH. **e** Scanning electron microscope images (left) and schematic diagrams of the capillary trailing (right). **f** Schematic diagram of polarized light detector (left). Angle-dependent photocurrent under different polarized light irradiation (right). Reproduced with permission from Ref. [55] Copyright 2017, Wiley–VCH

Nanostructure morphology provides a simple, effective, and low-cost way to improve performance and broaden the functionality of optoelectronic devices [8]. Geometric anisotropy is an additional approach for linearly polarized light detection, which requires an array size smaller than the operating wavelength [95, 96]. In particular, a series of linearly polarized photodetectors based on the 1D nanostructure of perovskite materials were successfully prepared [55, 86, 97, 98]. Inspired by the microstructure of butterfly wings, Li et al. [61] fabricated a 1D nano-grating structure atop a two-dimensional photonic crystal structure by synergistic spin-coating and nanoimprinting methods (Fig. 7a). The combination of the coupling effect of nano-grating structure and reflection of photonic crystals enhances the light capture capability of photodetectors, resulting in high-performance photodetectors with a responsivity of 12.67 A W^−1^ and detectivity of 3.22 × 10^13^ Jones. Meanwhile, the directionally aligned 1D nanograting array structure imparts a polarized light absorption property for the perovskite films, where the maximum absorption occurs with the polarized light parallel to the 1D array structure and the minimum absorption occurs with the polarized light perpendicular to the 1D array structure, yielding a high-performance linearly polarized photodetector with *g* of 1.6 (Fig. 7b). Furthermore, in situ encapsulated Moiré lattice-perovskite is fabricated by a double-grating structure PDMS templates (Fig. 7c) [88]. The prepared photodetectors exhibit enhanced photodetection performance with responsivity up to 1026.5 A W^−1^, which can be attributed to the Moiré lattice structure reducing reflection as well as enhancing absorption. At the same time, the stability of the photodetector has been greatly improved due to the encapsulation of the double-layer PDMS templates. In addition, the Moiré-lattice perovskite photodetector shows great sensitivity to polarized light. With the polarized light direction changing, the photocurrent shows a periodic variation, the maximum (minimum) photocurrent emerges when the polarized light is parallel (perpendicular) to the lattice direction, and the maximum polarization ratio reaches 9.1, revealing highly efficient detection capability for linearly polarized light (Fig. 7d).

Compared with the nanoimprinting technology, the fabrication of single-crystal nanowire arrays presents more advantages, such as higher crystallinity and less defect density, which are beneficial to the performance of photodetectors [71, 99–102]. Based on the sandwich assembly model, Wu et al. prepared nanowire arrays with strict alignment, precise locations, uniform sizes, and pure crystallographic orientation, which can be attributed to controlled nucleation as well as a slow growth process (Fig. 7e) [55]. The photodetector exhibits a large on/off ratio of nearly 10^3^ and responsivity of 1377 A W^−1^. The 1D array also presents the properties of linearly polarized light absorption because of its large aspect ratio and pure crystallographic orientation, ultimately yielding a high-performance linearly polarized photodetector with a polarization ratio of 2.6 (Fig. 7f). In addition, the stability of the microwire array exhibits significant improvement with only a 4% loss of photocurrent after one year in atmospheric conditions [103].

### Circular-Polarization Photodetectors

Circularly polarized light is a special elliptically polarized light whose electric vector rotates in a plane that is perpendicular to the direction of propagation and maintains a constant angular velocity [104, 105]. Compared to linearly polarized light detection, circularly polarized light detection has shown great promise in applications such as quantum computation, drug screening, optical communication, and magnetic recording [21, 22, 106, 107]. Although some progress has been made in circularly polarized photodetection, conventional circularly polarized photodetection requires additional quarter-wave plates and linear polarizers, leading to great difficulties in the integration and miniaturization of device applications [6, 108]. With the development of perovskite materials, a series of circularly polarized photodetectors based on perovskite materials have been fabricated [21, 46, 47, 109]. Currently, the realization of circularly polarized photodetectors for perovskite materials is largely based on the following mechanisms: (i) chiral crystal structure of perovskites; (ii) chiral perovskite/perovskite heterogeneous structure; (iii) perovskite/chiral nanoparticle heterogeneous structure; (iv) perovskite metasurfaces. The performance of representative circular-polarization photodetectors is summarized in Table 2.

**Table 2:** A summary of important figures of merit of representative perovskite circular-polarization photodetectors

Device structure	Materials	Polarization mechanism	*R* (A W^−1^)	D* (Jones)	Wavelength (nm)	Polarization ratio (*g*_res_)	References
Photoconductor	(*R*-/*S*-*α*-PEA)PbI_3_ film	Chiral crystal structure	0.797	7.1 × 10^11^	395	0.1	[21]
Photoconductor	[(*R*)-*β*-MPA]_2_MAPb_2_I_7_ film	Chiral crystal structure	1.1	2.3 × 10^11^	532	0.2	[110]
Photoconductor	(*R*-*β*-MPA)_4_AgBiI_8_ microwire arrays	Chiral crystal structure	5.2 × 10^–2^	3.9 × 10^11^	520	0.19	[109]
Photoconductor	(*R*-/*S*-*α*-PEA)_2_PbI_4_ nanowire arrays	Chiral crystal structure	47.1	1.2 × 10^13^	505	0.15	[47]
Photoconductor	(*R*-/*S*-C_5_H_14_N)PbI_3_ microwire arrays	Chiral crystal structure	2.6 × 10^–2^	2.2 × 10^11^	405	0.23	[58]
Photoconductor	[(*R*)-MPA]_2_MAPb_2_I_7_/ MAPbI_3_ heterocrystals	Heterogeneous structure	1.2 × 10^–3^	1.1 × 10^12^	520	0.67	[111]
Photoconductor	[(*R*)-MPA]_2_PbCl_4_/ Si heterostructure	Heterogeneous structure	2.0 × 10^–3^	1.2 × 10^12^	266	0.4	[112]
Photodiode	AuNPS/Cs_0.05_FA_0.5_MA_0.45_Pb_0.5_Sn_0.5_I_3_ film	Heterogeneous structure	0.24	2 × 10^13^	808	0.55	[51]
Photoconductor	CH_3_NH_3_PbI_3_ crystal	Geometry morphology	0.3	3 × 10^10^	700	−	[57]

#### Chiral Crystal Structure

**Fig. 8:**
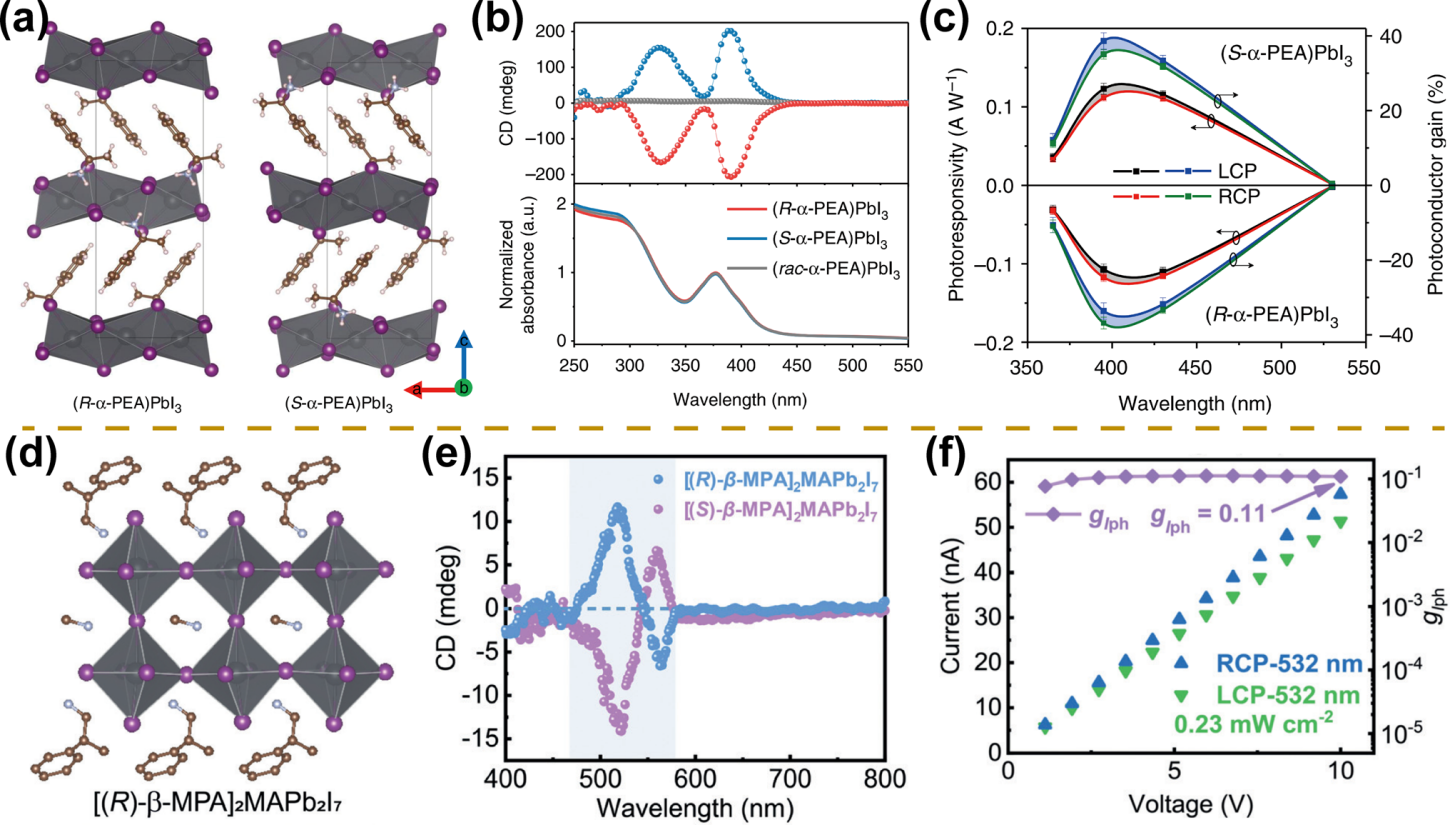
Circularly polarized light detection. **a** Schematic diagram of the crystal structures. **b** CD and absorbance spectra of 1D (*R*-/*S*-*rac*-MBA)PbI_3_ perovskite films. **c** Wavelength-dependent responsivities under the excitation of different circularly polarized light. Reproduced with permission from Ref. [21] Copyright 2019, Springer Nature. **d** Schematic diagram of the crystal structure, [(*R*)-*β*-MPA]_2_MAPb_2_I_7_. **e** CD spectra of 2D perovskite films. **f** Voltage-dependent currents under right-handed circularly polarized (RCP) and left-handed circularly polarized (LCP) light at the wavelengths of 532 nm. Reproduced with permission from Ref. [110] Copyright 2020, Wiley–VCH

The diverse structures, large absorption coefficients, and solution-processing properties of organic–inorganic hybrid perovskite have significantly extended the application range of devices from spintronics and memory devices to nonlinear optical devices [6, 113–118]. Among them, circularly polarized photodetectors based on chiral perovskites have also made rapid development [58, 109, 119–122]. Jooho Moon et al. first synthesized chiral 2D perovskites named (*R*-/*S*-MBA)_2_PbI_4_ and demonstrated their strong circular dichroic absorption properties with opposite signal values for the left- and right-handed perovskites [123]. Furthermore, Tang et al. synthesized chiral 1D perovskite (*R*-/*S*-MBA)PbI_3_ that exhibits strong circularly polarized absorption properties attributed to the chiral transfer from chiral ammoniums to 1D perovskite octahedral framework (Fig. 8a, b) [21]. The wavelength-dependent responsivity under different circularly polarized light irradiation indicates that chiral perovskite possesses the property of circularly polarized light detection (Fig. 8c). High-performance circularly polarized photodetectors are demonstrated with a *g*_res_ of 0.1, a responsivity of 797 mA W^−1^, detectivity of 7.1 × 10^11^ Jones, and long-term environmental stability. Yuan et al. [110] further fabricated a higher layer number of 2D chiral perovskites [(*R*)-*β*-MPA]_2_MAPb_2_I_7_ that exhibit circular dichroic absorption behavior (Fig. 8d, e). Under 532 nm circularly polarized illumination, higher photocurrents are obtained under right-hand circularly polarized illumination compared to left-hand circularly polarized illumination, demonstrating the superior circularly polarized light detection properties of the device (Fig. 8f). The high responsivity of 1.1 A W^−1^, *g*_res_ of 0.2, and excellent mechanical stability are confirmed for this photodetector.

Despite the above lead-based perovskite photodetectors exhibiting excellent performance for circularly polarized light detection, perovskites usually contain toxic lead elements, which limits the commercial application of the devices [16]. For this reason, lead-free perovskite circularly polarized photodetectors have been developed [108, 123]. Zhao et al. prepared pure oriented chiral double perovskite ((*R*-/S-*β*-MPA)_4_AgBiI_8_) microwire arrays by capillary-bridge confined assembly technique [109]. The microwire arrays exhibit excellent circular dichroic absorption properties as well as high environmental stability, yielding outstanding circularly polarized photodetector with a maximum *g*_res_ of 0.19, responsivity exceeding 52 mA W^−1^, and detectivity exceeding 3.9 × 10^11^ Jones.

#### Perovskite/Perovskite Heterogeneous Structure

Except for circularly polarized photodetection based on the single-component perovskites, the two-component perovskite/perovskite heterojunction shows better circular polarization sensitivity due to the reduced electron–hole recombination rate [111, 112, 125]. Circularly polarized photodetectors that cover the ultraviolet to the near-infrared region are achieved through the development of chiral perovskite heterojunctions [111, 112, 125]. For ultraviolet region detection, perovskite/silica heterojunctions were constructed, showing superior circular polarization detection performance at 266 nm with *g*_res_ up to 0.4 [111]. In the visible region, Luo et al. fabricated 2D/3D perovskite heterostructure crystals ([(*R*)-MPA]_2_MAPb_2_I_7_/MAPbI_3_) by solution-processed heteroepitaxy [111]. The sharp heterogeneous interface facilitates the formation of the built-in electric field, thus promoting the separation of charge carriers as well as the chiral transfer. Circularly polarized photodetectors finally achieve impressively large *g*_res_ up to 0.67, a responsivity value of 1.2 mA W^−1^, and a detectivity value of 1.1 × 10^12^ Jones at 520 nm.

**Fig. 9:**
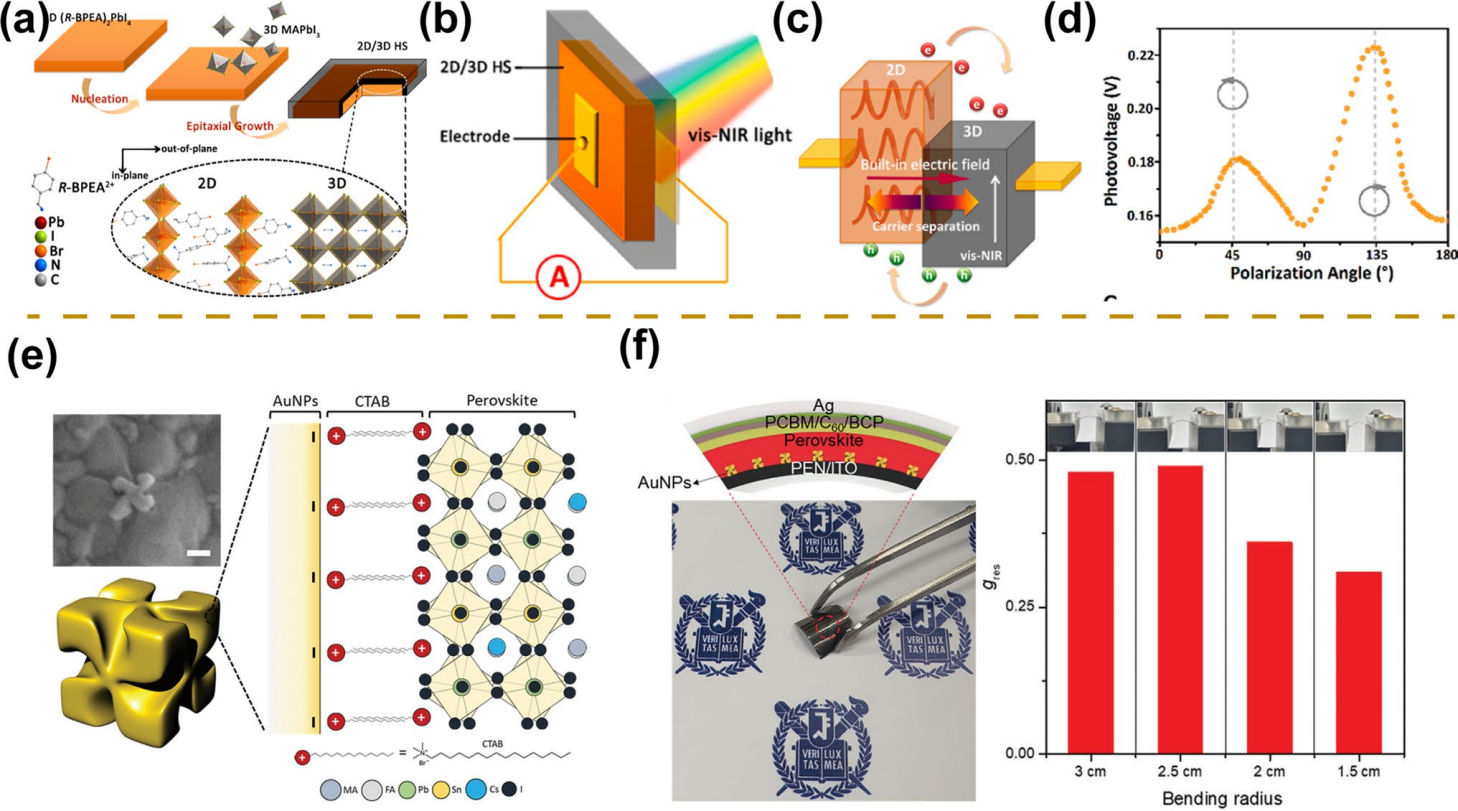
Circularly polarized light detection. **a** Preparation process of perovskite heterojunction. **b** Schematic diagram of circularly polarized light detection based on perovskite heterojunction. **c** Diagram of the working mechanism for a heterojunction photodetector. **d** Angle-dependent photocurrents under circularly polarized light irradiation with the different rotation angles of a *λ*/4 plate. Reproduced with permission from Ref. [125] Copyright 2022, American Chemical Society. **e** Schematic diagram of the interaction between AuNPs and perovskite (right) and its SEM image (left). **f** Schematic diagram of a flexible circularly polarized photodetector and its photograph (left). *g*_res_ at different bending radii (right). Reproduced with permission from Ref. [51] Copyright 2022, Wiley–VCH

Since the cutoff wavelength edge of chiral perovskite is commonly located in the ultraviolet and visible regions, it is difficult to achieve near-infrared circularly polarized light detection. To achieve the detection of near-infrared circularly polarized light, the formation of chiral perovskite heterojunction is a valuable approach. Based on the discussion above, heterostructures of 3D perovskite epitaxially grown on the surface of 2D perovskite were successfully developed by the solution-processed epitaxial growth method [125]. (*R*-BPEA)_2_PbI_4_/MAPbI_3_ heterojunction photodetector enables efficient near-infrared circularly polarized photodetection with large *g*_res_ up to 0.25, on/off switching ratio of 10^5^, and detectivity value of 1.1 × 10^10^ Jones at 785 nm (Fig. 9a–d). Overall, perovskite heterojunction photodetectors exhibit enhanced performance for circularly polarized light detection, which has greatly boosted their development.

#### Perovskite/Chiral Nanoparticle Heterogeneous Structure

Despite the significant progress of circularly polarized photodetectors based on chiral perovskites and their heterojunction have been realized, their low-dimensional structures sacrifice the conductivity of perovskites, thus limiting the performance improvement of the detector. To ensure both the electrical conductivity of perovskites and the property of circularly polarized light absorption, chiral nanoparticles embedded in a perovskite matrix are a promising way [51]. Joon et al. prepared chiral gold nanoparticles embedded perovskite films, named AuNPs/ Cs_0.05_FA_0.5_MA_0.45_Pb_0.5_Sn_0.5_I_3_, exhibiting good circular dichroic absorption properties which can be attributed to the plasmon resonance shift of chiral plasmonic AuNPs (Fig. 9e). In addition, the incorporation of chiral plasmonic AuNPs did not sacrifice the optoelectronic properties of the perovskite films. Based on nanoparticle-embedded perovskite films, superior near-infrared circularly polarized photodetectors are developed with large *g*_res_ up to 0.55. Ultimately, the flexible circularly polarized photodetector is demonstrated with excellent mechanical stability and reproducibility (Fig. 9f). In addition to chiral plasmonic AuNPs, excellent circularly polarized photodetection may be achieved by embedding other chiral nanoparticles. Lead-free circularly polarized photodetectors can also be achieved in this way.

#### Perovskite Metasurface Structure

Metasurface is a planar artificial material consisting of subwavelength structural units that exhibits an excellent ability to control the wavefront of electromagnetic waves, and there has been a rapid development of perovskite metasurface-based optoelectronic devices in recent years, such as vortex lasers, polarized light-emitting transistors, and circularly polarized photodetectors [57, 126–131]. The introduction of additional functionality for optoelectronic devices can be attributed to the sub-wavelength structure of the metasurface that can generate strong light-matter interactions, thus affecting the polarization, amplitude, and phase of the light [126, 127]. The preparation of perovskite metasurface is commonly based on electron beam etching, ion beam etching, and laser etching [40, 57, 78, 129, 131]. Wang et al. fabricated periodic antenna structures on the surface of perovskite single crystals using focused ion beams, forming a perovskite metasurface that exhibits absorption differences for circularly polarized light with different polarization states [57]. The difference in absorption for different circular polarizations of light can be attributed to the mirror asymmetry of the antenna elements. Furthermore, circularly polarized photodetectors are successfully fabricated based on perovskite metasurface, opening up new avenues for the development of integrated optoelectronics.

### Stokes-Parameter Photodetectors

**Fig. 10:**
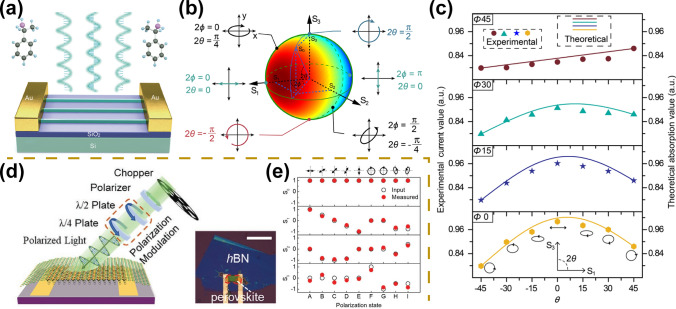
Stokes-parameter light detection. **a** Schematic diagram of the Stokes-parameter photodetector based on chiral perovskite nanowire arrays. **b** Theoretical absorption intensity for different Stokes parameters presented on the Poincare-spheré. **c** Experimental measurements and theoretically derived values for different states of polarized light. Reproduced with permission from Ref. [47] Copyright 2021, American Chemical Society. **d** Schematic diagram of the full-Stokes polarimeter based on chiral 2D perovskite single crystals. **e** Experimental measurements and input values for polarized light with different polarization states. Reproduced with permission from Ref. [62] Copyright 2021, Wiley–VCH

Compared with linearly and circularly polarized photodetectors, stokes-parameter photodetectors present more important advantages because of their ability to detect arbitrarily polarized light, including elliptical polarization, linear polarization, and circular polarization, which can greatly reduce the size and accelerate the miniaturization and integration of optoelectronic devices [132]. In recent years, stokes-parameter photodetectors based on perovskite materials have also been investigated [47, 62]. For example, Zhao et al. fabricated chiral perovskite nanowire arrays using the capillary-bridge confined assembly method (Fig. 10a) [47]. In synergy with their molecular-scale circularly polarized light absorption and nanometer-scale large aspect ratio possessing linearly polarized light absorption, the linearly polarized as well as circularly polarized light detection is achieved simultaneously. Furthermore, they extracted anisotropic absorption coefficients for both linearly and circularly polarized light of chiral perovskite nanowire devices and calculated the normalized absorption coefficients represented by the Stokes parameter, showing a remarkable dependence (Fig. 10b). The authors studied four typical polarization ellipse orientations. The measured photocurrents coinciding with the calculated absorption coefficients demonstrate the successful fabrication of the Stokes photodetector (Fig. 10c). Stokes-parameter photodetectors also exhibit outstanding photodetection performance with a responsivity of 47.1 A W^−1^ and a specific detectivity of 1.24 × 10^13^ Jones. In addition, Li et al. demonstrated another full-Stokes polarimeter by synergizing the anisotropy of the chiral perovskite single-crystal structure and its circular polarization absorption properties (Fig. 10d) [62]. Consistent results between experimental and theoretical values demonstrate the successful preparation of a full-Stokes polarimeter (Fig. 10e).

### Spectrum-Sensitive Photodetectors

In addition to the detection of polarized light, spectral detection is also extremely important because of promising applications in the fields of imaging, sensing, autonomous driving, robotics, and optical communications [23, 24, 26, 133, 134]. However, conventional spectral detectors require complex optical prisms or interference filters for spectral recognition, which hinders the miniaturization and integration of optoelectronic devices. In this section, we will focus on filter-free perovskite spectral detectors, including narrow-band photodetectors and spectrometers.

#### Narrow-Band Photodetectors

Narrow-band photodetectors are a category of photodetectors that can selectively detect specific wavelengths [11, 81, 135–137]. According to the working mechanism, the implementation of narrow-band photodetectors is mainly based on four routes [138]. The synergy of broadband photodetectors and bandpass filters for narrow-band light detection is the first route, but the high cost and its complex integration system limit the commercialization [139]. The second route is the synthesis of narrow-band absorbing materials [140]. Although they can achieve narrow-band light detection, their spectrum is usually located in visible-blind or solar-blind regions, and the preparation of narrow-band devices in visible or near-infrared regions is more challenging. The third route is based on the selective design of optical structure devices for enhancing light absorption at specific wavelengths, but it is limited by the narrow spectral operating range and its complex processing [141]. In contrast to the above routes, the construction of thick perovskite thin film or bulk single-crystal devices shows remarkable properties, such as low-cost solution processing, continuously tunable band gap, and simplified device structure [140, 142].

**Fig. 11:**
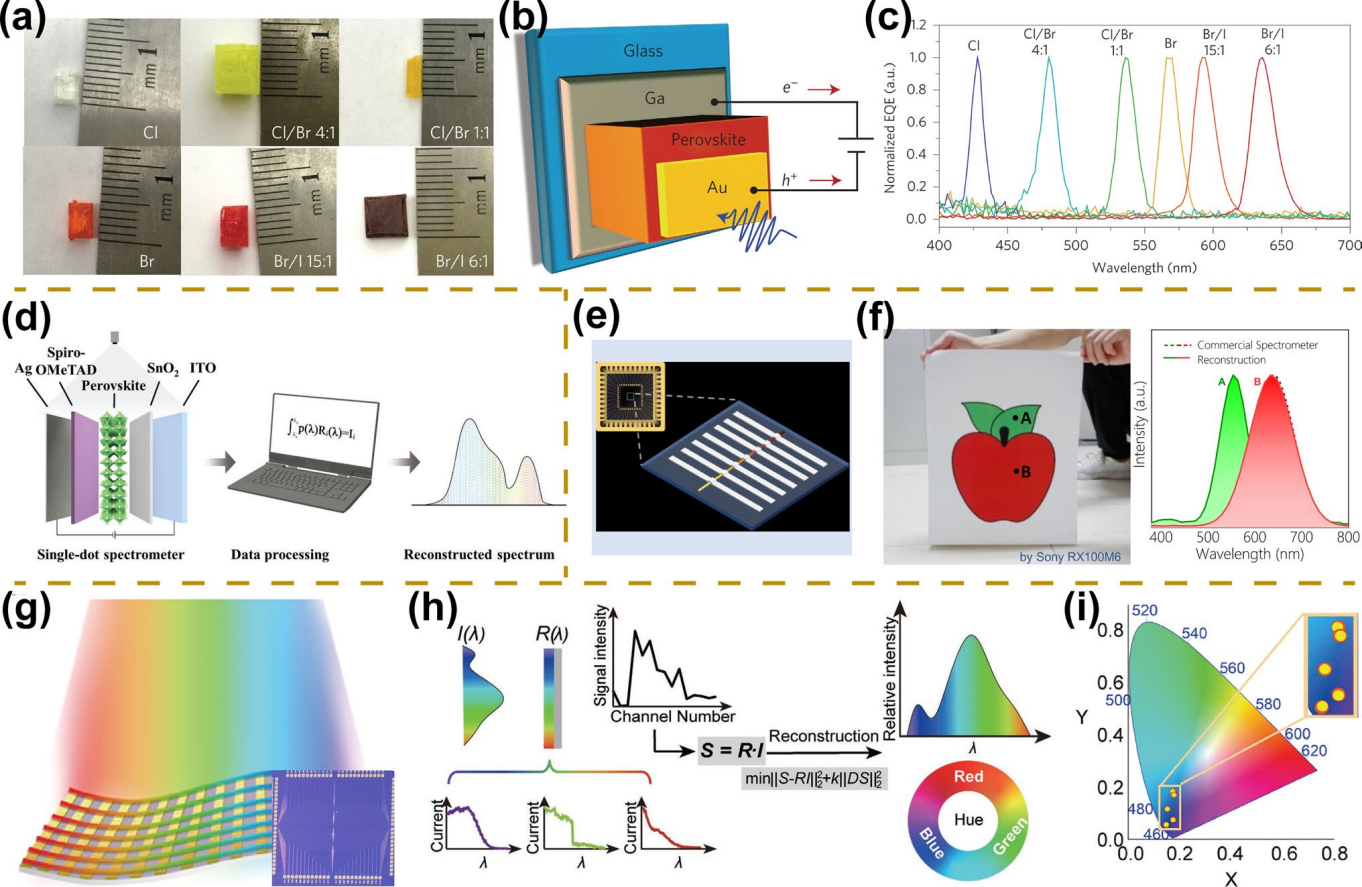
Spectrum-sensitive photodetectors. **a** Photographs of perovskite single crystals with different halide compositions. **b** Schematic diagram of narrow-band photodetectors. **c** Normalized external quantum efficiency pattern of perovskite single crystals with different halogen compositions, demonstrating the capability of narrow-band photodetection. Reproduced with permission from Ref. [138] Copyright 2015, Springer Nature. **d** Schematic diagram and working mechanism of a single-dot perovskite spectrometer. Reproduced with permission from Ref. [27] Copyright 2022, Wiley–VCH. **e** Schematic diagram of a perovskite microwire multispectral detector. **f** Photograph of spectral reconstruction (left) and their spectral reconstruction results based on perovskite microwire multispectral detectors. Reproduced with permission from Ref. [65] Copyright 2021, Wiley–VCH. **g** Schematic diagram of perovskite flexible spectrometer based on gradient bandgap-tunable perovskite microwire arrays. The inset is a photograph of the integrated spectrometer. **h** Schematic diagram and working mechanism of perovskite flexible spectrometer. **i** Reconstructed spectra of similar blue color within the CIE chromaticity diagram demonstrate the ability to recognize similar colors. Reproduced with permission from Ref. [48] Copyright 2023, Wiley–VCH

Narrow-band photodetectors are successfully prepared based on thick film or large single crystals of perovskites. For example, Huang et al. fabricated perovskite single-crystal narrow-band photodetectors that can detect spectra with a full width at a half-maximum of less than 20 nm (Fig. 11a–c) [138]. By adjusting the halogen composition of the perovskite single crystal, the response spectrum of the narrow-band photodetector can be tuned continuously from blue to red. The structure of a narrow-band photodetector mainly consists of a Ga cathode, a semi-transparent Au anode, and a perovskite single crystal (Fig. 11b). The mechanism of narrow-band photodetection based on bulk single crystals can be attributed to the fact that only long-wavelength light can generate photocarriers collected by the electrodes due to their high optical penetration depth, whereas short-wavelength light is lost before they are transported to the electrode due to strong surface-charge recombination.

#### Perovskite Spectrometer

In addition to the fabrication of narrow-band photodetectors, perovskite spectrometers enable precise identification of the spectrum of incident light which has important applications in traditional as well as emerging fields [20, 143]. Li et al. first prepared a self-powered perovskite spectrometer by synergizing gradient bandgap-tunable perovskite films and a reconfiguration algorithm [146]. Perovskite spectrometers exhibit excellent optoelectronic performance with a fast response time of 200 ns, high external quantum efficiency of up to 94%, and spectral resolution of up to 80 nm. Based on gradient bandgap-tunable perovskite films, Ding et al. also fabricated perovskite spectrometers and assembled flexible devices for chemical/bio-sensing applications [143]. In addition, Li et al. fabricated a micrometer-level single-dot perovskite film spectrometer possessing a footprint of 440 × 440 µm^2^ based on the mechanism of the photogain manipulation at various bias voltages resulting in ion redistribution within the perovskite film (Fig. 11d) [27]. Compared to previous perovskite spectrometers, single-dot perovskite spectrometer exhibits superior spectral discrimination performance with spectral resolution up to 5 nm.

Despite the significant progress of perovskite thin film spectrometers, a lot of defects within perovskite thin films will cause higher dark currents in the devices, thus limiting the sensitivity of perovskite spectrometers, which is detrimental to their practical applications [48, 65]. Based on the above reasons, the research of single crystal perovskite microwire spectrometer seems to be important. Zeng et al. developed perovskite multispectral detectors based on a gradient bandgap MAPbX_3_ microwire with a diameter of 30 μm and a length of 4 mm (Fig. 11e) [65]. The optical bandgap of gradient bandgap perovskite microwire can cover 2.96 to 1.68 eV, corresponding to the Cl-rich to I-rich region. High-performance multispectral detectors based on high-quality gradient bandgap perovskite microwire are prepared with a spectral resolution of 25 nm, responsivity over 20 mA W^−1^, − 3 dB bandwidth of 450 Hz, and low noise currents less than 1.4 × 10^−12^ A Hz^−0.5^ (Fig. 11f). Furthermore, Wu et al. prepared gradient bandgap-tunable perovskite microwire arrays by synergizing the capillary liquid bridge assembly technique and its anion exchange engineering, yielding the integrated perovskite spectrometer (Fig. 11g, h) [48]. The high crystallinity and the pure crystallographic orientation of the microwire arrays enable a high-performance perovskite spectrometer with responsivities of 10^3^ A W^−1^, detectivities of 10^15^ Jones, and a spectral resolution of 10 nm covering 405–760 nm. Flexible color-cognitive devices have also been developed to recognize similar colors for the correction of color blindness patients (Fig. 11i). The performance of representative perovskite spectrometers is summarized in Table 3.

**Table 3:** A summary of important figures of merit of representative perovskite spectrometers

Device structure	Materials	*R* (A W^−1^)	D* (Jones)	Spectral range (nm)	Spectral resolution (nm)	References
Photoconductor	KMAPbCl_x_Br_3-x_ film	–	–	450–780	80	[144]
Photoconductor	MAPbX_3_ microwire	0.02	3.5 × 10^9^	450–790	25	[65]
Photoconductor	CsPbX_3_ quantum dot	–	–	400–700	10	[145]
Photodiode	Cs_0.05_MA_0.15_FA_0.8_PbI_0.85_Br_0.15_ film	49	10^11^	350–750	5	[27]
Photoconductor	MAPbX_3_ microwire arrays	10^3^	10^15^	405–760	10	[48]

### Angle-Sensing Photodetectors

Angle-sensing photodetectors refer to a class of detector devices that are sensitive/insensitive to the direction of incident light [19, 54, 66, 67, 146, 147]. The conventional implementation of angle-sensing photodetectors relies on the physical combination of photodetectors with large optical lenses, resulting in bulky and complex device structures, which is detrimental to the development of miniaturized and integrated devices. To achieve the angle-sensing photodetectors, a series of perovskite-based devices have been investigated. According to their functions, they can be divided into two main categories: (i) omnidirectional photodetectors which are insensitive to the incident angle of light; (ii) angle-recognition photodetectors which are sensitive to the incident angle of light.

#### Omnidirectional Photodetectors

**Fig. 12:**
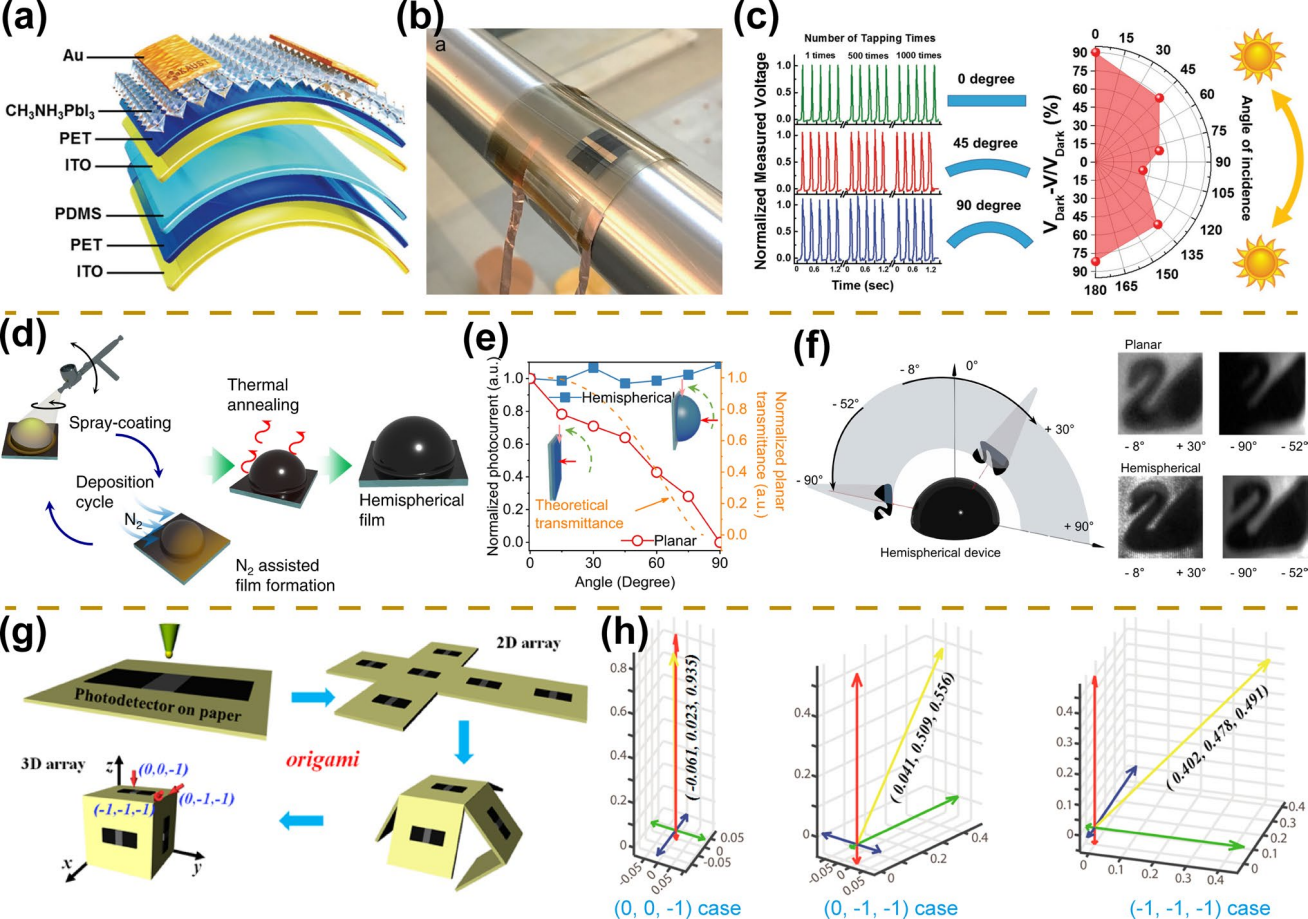
Angle-sensing photodetectors. **a** Schematic diagram of a flexible transparent perovskite photodetector. **b** Photographs of flexible transparent perovskite photodetector on the curved surface. **c** Device performance at different bending angles (left) and different incidence angles (right). Reproduced with permission from Ref. [66] Copyright 2017, Wiley–VCH. **d** Schematic diagram of the preparation of hemispherical photodetector. **e** Normalized photocurrent at different incidence angles. **f** Schematic diagram of imaging at different incidence angles (left) and imaging results of hemispherical and planar devices (right). Reproduced with permission from Ref. [67] Copyright 2022, Springer Nature. **g** Schematic diagram of the fabrication of a three-dimensional configured photodetector. **h** Device corresponds to different cartesian coordinates at different incidence angles of light. Reproduced with permission from Ref. [54] Copyright 2017, American Chemical Society

The 360° spatial light detection has boosted the development of imaging, sensing, medical, and communication fields. Compared to planar photodetectors that can only recognize incident light at a specific angle, 360° omnidirectional photodetectors show great advantages for land-based/underwater internet systems, driverless navigation, intraoperative navigated surgery, and robotics [66, 67, 147]. For the preparation of omnidirectional photodetectors, flexible-based, fiber-based, and hemispherical-type perovskite photodetectors are developed. For example, Leung et al. prepared flexible perovskite photodetectors that exhibited excellent omnidirectional detection performance, which can be attributed to their flexible transparent device structure (Fig. 12a, b) [66]. The devices also exhibit excellent bending performance at different bending angles (Fig. 12c). The voltage value presents a slight variation at different incidence angles indicating its omnidirectional detection capability. Inspired by the fish-eye and compound-eye architecture, Wei et al. prepared a hemispherical photodetector by spray-coating technology (Fig. 12d) [67]. High-performance omnidirectional angle detectors are prepared by controlling the concentration of the perovskite solution and the spray-coating cycles. The lens-free hemispherical photodetector device shows a stable photocurrent and imaging with a wide incidence angle of 180° (Fig. 12e, f). In addition, Zeng et al. prepared wearable fiber-based omnidirectional UV photodetectors by dip-coating method [147]. Compared to flexible planar devices and their hemispherical detectors, fiber-based detectors show superior omnidirectional detection performance. Unnoticeable changes in device responsivities are demonstrated under different incidence angles of light. The fabrication of omnidirectional photodetectors will greatly facilitate emerging fields including intelligent recognition and intraoperative navigation surgery.

#### Angle-Recognition Photodetectors

Compared to omnidirectional photodetectors, angle-recognition photodetectors also facilitate the development of emerging fields such as artificial intelligence and autonomous driving [19, 54, 146]. Currently, the fabrication of angle-recognition photodetectors mainly relies on the physical combination of multiple detectors with additional prisms, resulting in high cost and large size of the devices. In addition, the preparation of perovskite-based angle-recognition photodetectors is still scarce. Inspired by ingenious origami, Yan et al. prepared the first three-dimensional configured photodetector that exhibits excellent discrimination ability for spatial light direction (Fig. 12g) [54]. The structure of the device mainly consists of a paper substrate, graphite electrodes, and perovskite materials, which have an extremely low cost. The excellent deformation properties for paper-based device structures facilitate the preparation of three-dimensional structured devices. Compared with conventional photodetectors, the three-dimensional photodetector can determine the arbitrary direction of light in space, which is confirmed by signal processing for three different coordinates of the incident light (Fig. 12h).

### Self-powered Photodetector

**Fig. 13:**
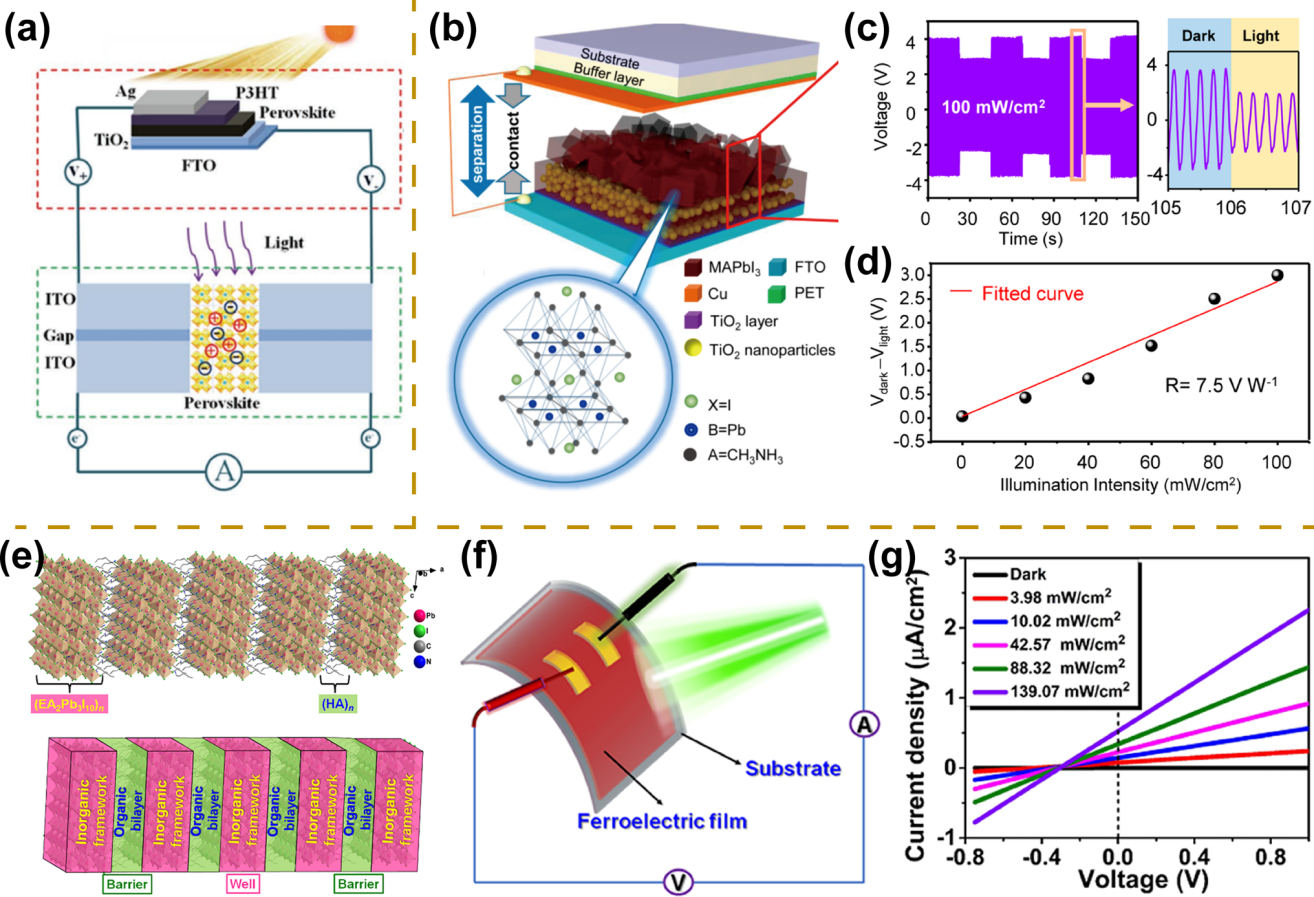
Self-powered photodetector. **a** Schematic diagram of self-powered photodetector through integration with a perovskite solar cell. Reproduced with permission from Ref. [149], Copyright 2017, Wiley–VCH. **b** Schematic diagram of self-powered photodetectors based on the triboelectric effect of perovskite materials. **c** Voltage variation under repeated illumination with a power of 100 mV cm^−2^. **d** Voltage variation under different light intensity irradiation. Reproduced with permission from Ref. [150], Copyright 2015, American Chemical Society. **e** Schematic diagram of the crystal structure of perovskite ferroelectric material. **f** Schematic diagram of flexible self-powered broadband photodetectors. **g** *I–V* curves under different light intensity irradiation with short-circuit photocurrent density of 0.55 μA cm^−2^. Reproduced with permission from Ref. [69] Copyright 2022, American Chemical Society

Commercialization of photodetectors requires detectors with low power consumption and excellent photodetector performance [148]. So far, although great efforts have been achieved in multifunctional perovskite photodetectors with high sensitivity and fast response speed, relatively limited work has been focused on low-power or self-powered devices. Self-powered photodetector refers to a category of devices that can achieve photoelectric conversion without external power sources, showing great promise in miniaturization, integration, and flexible wireless sensing [28, 148]. To realize the fabrication of self-powered detectors, device structure optimization, and composition engineering are developed, such as integration with external energy harvesting/storage units, triboelectrification/piezoelectricity, p–n junction, Schottky junction, and bulk photovoltaic effects within ferroelectric materials [69, 149]. In 2016, Li et al. first prepared an all-perovskite self-powered photodetector through integration with a perovskite solar cell (Fig. 13a) [149]. Based on the energy supply from the solar cell, the photodetector exhibits stable performance with a drive voltage of less than 1 V. Despite the achievement of self-powered photodetectors, their complex structures, and laborious fabrication processes limit their practical application in the future. In contrast to complex external integration, self-powered photodetectors powered by triboelectrification exhibit significant advantages, such as simpler structures and superior performance. Zhu et al. prepared self-powered photodetectors by combining the optoelectronic properties and triboelectric effect of perovskite materials [150]. The main configuration of the device consists of an FTO substrate, titanium dioxide transport layers, a perovskite active layer, a copper layer, and an elastic buffer layer (Fig. 13b). The surface triboelectric density of perovskite composite films changes significantly under solar illumination (Fig. 13c, d). Based on this device structure, the self-powered photodetector exhibits excellent performance with a high responsivity of 7.5 A W^−1^, a fast response time of 80 ms, and a wide spectral response range.

Compared to the above-mentioned devices, which usually require complex interface engineering as well as fabrication processes, self-powered photodetectors based on perovskite ferroelectric materials present a simpler structure and excellent performance [151]. The built-in electric field intensity of single-phase perovskite ferroelectric materials is mainly associated with their spontaneous polarization intensity, which is an order of magnitude higher than that of conventional device structures, such as the Schottky barrier and p–n junction [152, 153]. To achieve the fabrication of narrower bandgap perovskite ferroelectric materials which can enhance their visible light absorption properties, Luo et al. prepared a new biaxial perovskite ferroelectric material ((isopentylammonium)_2_(ethylammonium)_2_Pb_3_I_10_) that exhibits large spontaneous polarization of 5.2 μC cm^−2^ as well as narrow bandgap of 1.80 eV (Fig. 13e). The bulk photovoltaic and photoelectric effects of ferroelectric materials endow the devices with excellent self-propelled detection performance with a high current density of 1.5 μA cm^−2^, a large switching ratio of 10^5^, and a wide spectral response range of 365–670 nm (Fig. 13f, g) [151]. Furthermore, they prepared perovskite ferroelectric material (HA_2_EA_2_Pb_3_I_10_) with eight equivalent polarization directions, presenting in-plane spontaneous polarization of 1.8 μC cm^−2^. Based on this material, flexible self-powered broadband photodetectors are successfully prepared with a current density of 0.55 μA cm^−2^ under 637 nm illumination [69]. The performance of representative perovskite self-powered photodetectors is summarized in Table 4.

**Table 4:** A summary of important figures of merits of representative perovskite self-powered photodetectors

Device structure	Materials	Self-powered type	*R* (A W^−1)^	D* (Jones)	Wavelength (nm)	References
Photoconductor	CH_3_NH_3_PbI_3_ film	Integration with solar cell	0.16	–	750	[149]
Photoconductor	CH_3_NH_3_PbI_3_ film	Triboelectrification	–	–	–	[150]
Photoconductor	CH_3_NH_3_PbI_3_ crystal	Triboelectrification	7.92	–	–	[154]
Photoconductor	CH_3_NH_3_PbI_3_ film	Triboelectrification	0.418	1.22 × 10^13^	–	[66]
Photodiode	CH_3_NH_3_PbI_3_ film	p–n junction	0.563	2.15 × 10^13^	800	[153]
Photodiode	CsPbBr_3_ film	p–n junction	0.44	1.9 × 10^13^	405	[155]
Photodiode	CsPbBr_3_ quantum dot	p–n junction	10.1	9.35 × 10^13^	405	[156]
Photoconductor	CH_3_NH_3_PbI_3_ crystal	Schottky junction	2.0 × 10^–3^	1.4 × 10^10^	532	[152]
Photoconductor	CsPbBr_3_ crystal	Schottky junction	2.8 × 10^–2^	1.7 × 10^11^	550	[157]
Photoconductor	[(*R*)-*β*-MPA]_4_AgBiI_8_ crystal	Bulk photovoltaic effects	2.2 × 10^–5^	1.2 × 10^7^	520	[124]
Photoconductor	HA_2_EA_2_Pb_3_I_10_ film	Bulk photovoltaic effects	–	–	637	[69]

## Challenges and Perspectives

In this review, we first describe crystal structure design and micro/nano-scale morphology manipulation of perovskite materials. Then we summarize the structural categories and figure of merits for photodetectors. Finally, we systematically overview the functional photodetectors based on perovskite materials, mainly including polarization light detectors, spectral detectors, angle-sensing detectors, and self-powered detectors. Although significant progress has been achieved in perovskite-based functional photodetectors, many challenges remain to be addressed. The main development perspectives are proposed.

(i) Miniaturized and integrated perovskite photodetectors need to be further investigated. In particular, the integration processing of perovskite subwavelength photodetectors needs to be realized. Non-destructive processing techniques for subwavelength perovskite arrays need to be developed.
(ii) The performance of perovskite multifunctional detectors strongly depends on the crystallinity and orientation of perovskite materials. Therefore, single-crystal perovskite functional detectors need to be fabricated.
(iii) To minimize the size and structural complexity of the device. Simultaneous implementation of polarized light, spectral and angle detection within a single perovskite multifunction detector is a promising research direction.
(iv) The limited band gap of perovskite material results in the preparation of multifunctional photodetectors mainly focusing on the visible light region, and infrared light detection is less studied. To realize the fabrication of infrared functional photodetectors, the nonlinear optical absorption effect is an effective way.
(v) Most of the multifunctional photodetectors are prepared based on lead-based perovskite material, the toxicity within perovskite is not conducive to large-area commercialization. Therefore, non-lead perovskite multifunctional photodetectors need to be developed, such as tin-based perovskite, double perovskite, etc.
(vi) To meet the development of flexible wearable devices, flexible multifunctional photodetectors also need to be developed.

## References

[1] Kelley SO, Mirkin CA, Walt DR, Ismagilov RF, Toner M (2014). Advancing the speed, sensitivity and accuracy of biomolecular detection using multi-length-scale engineering. Nat. Nanotechnol..

[2] Casalino M, Coppola G, De La Rue RM, Logan DF (2016). State-of-the-art all-silicon sub-bandgap photodetectors at telecom and datacom wavelengths. Laser Photonics Rev..

[3] Chen H, Liu H, Zhang Z, Hu K, Fang X (2016). Nanostructured photodetectors: from ultraviolet to terahertz. Adv. Mater..

[4] Jansen-van Vuuren RD, Armin A, Pandey AK, Burn PL, Meredith P (2016). Organic photodiodes: the future of full-color detection and image sensing. Adv. Mater..

[5] Wang H, Kim DH (2017). Perovskite-based photodetectors: materials and devices. Chem. Soc. Rev..

[6] Long GK, Sabatini R, Saidaminov MI, Lakhwani G, Rasmita A (2020). Chiral-perovskite optoelectronics. Nat. Rev. Mater..

[7] Wang F, Zou X, Xu M, Wang H, Wang H (2021). Recent progress on electrical and optical manipulations of perovskite photodetectors. Adv. Sci..

[8] Wang HP, Li S, Liu X, Shi Z, Fang X (2021). Low-dimensional metal halide perovskite photodetectors. Adv. Mater..

[9] Ahmadi M, Wu T, Hu B (2017). A review on organic-inorganic halide perovskite photodetectors: device engineering and fundamental physics. Adv. Mater..

[10] Cubero S, Aleixos N, Moltó E, Gómez-Sanchis J, Blasco J (2010). Advances in machine vision applications for automatic inspection and quality evaluation of fruits and vegetables. Food Bioprocess. Technol..

[11] Xue J, Zhu Z, Xu X, Gu Y, Wang S (2018). Narrowband perovskite photodetector-based image array for potential application in artificial vision. Nano Lett..

[12] ElHamdani S, Benamar N, Younis M (2020). Pedestrian support in intelligent transportation systems: challenges, solutions and open issues. Transp. Res. Part C Emerg. Technol..

[13] Chen G, Qiu Y, Gao H, Zhao Y, Feng J (2020). Air-stable highly crystalline formamidinium perovskite 1D structures for ultrasensitive photodetectors. Adv. Funct. Mater..

[14] Li Z, Hong E, Zhang X, Deng M, Fang X (2022). Perovskite-type 2D materials for high-performance photodetectors. J. Phys. Chem. Lett..

[15] Xu W, Huang W, Wu X, Wei X, Meng F (2021). 2D perovskite single crystals for photodetectors: from macro-to microscale. Phys. Status Solidi RRL.

[16] Zhang Y, Ma Y, Wang Y, Zhang X, Zuo C (2021). Lead-free perovskite photodetectors: progress, challenges, and opportunities. Adv. Mater..

[17] Hao D, Zou J, Huang J (2019). Recent developments in flexible photodetectors based on metal halide perovskite. InfoMat.

[18] Miao J, Zhang F (2019). Recent progress on highly sensitive perovskite photodetectors. J. Mater. Chem. C.

[19] Yi S, Zhou M, Yu Z, Fan P, Behdad N (2018). Subwavelength angle-sensing photodetectors inspired by directional hearing in small animals. Nat. Nanotechnol..

[20] Yang Z, Albrow-Owen T, Cui H, Alexander-Webber J, Gu F (2019). Single-nanowire spectrometers. Science.

[21] Chen C, Gao L, Gao W, Ge C, Du X (2019). Circularly polarized light detection using chiral hybrid perovskite. Nat. Commun..

[22] Huang PJ, Taniguchi K, Miyasaka H (2019). Bulk photovoltaic effect in a pair of chiral-polar layered perovskite-type lead iodides altered by chirality of organic cations. J. Am. Chem. Soc..

[23] Hartmann W, Varytis P, Gehring H, Walter N, Beutel F (2020). Broadband spectrometer with single-photon sensitivity exploiting tailored disorder. Nano Lett..

[24] Meng J, Cadusch JJ, Crozier KB (2020). Detector-only spectrometer based on structurally colored silicon nanowires and a reconstruction algorithm. Nano Lett..

[25] Liu L, Yang Y, Wang Y, Adil MA, Zhao Y (2022). Building supramolecular chirality in bulk heterojunctions enables amplified dissymmetry current for high-performing circularly polarized light detection. ACS Mater. Lett..

[26] Yang Z, Albrow-Owen T, Cai W, Hasan T (2021). Miniaturization of optical spectrometers. Science.

[27] Guo L, Sun H, Wang M, Wang M, Min L (2022). A single-dot perovskite spectrometer. Adv. Mater..

[28] Perumal Veeramalai C, Feng S, Zhang X, Pammi SVN, Pecunia V (2021). Lead–halide perovskites for next-generation self-powered photodetectors: a comprehensive review. Photonics Res..

[29] Shi D, Adinolfi V, Comin R, Yuan M, Alarousu E (2015). Low trap-state density and long carrier diffusion in organolead trihalide perovskite single crystals. Science.

[30] Stranks SD, Snaith HJ (2015). Metal-halide perovskites for photovoltaic and light-emitting devices. Nat. Nanotechnol..

[31] Tsai H, Nie W, Blancon JC, Stoumpos CC, Asadpour R (2016). High-efficiency two-dimensional ruddlesden-popper perovskite solar cells. Nature.

[32] Blancon JC, Tsai H, Nie W, Stoumpos CC, Pedesseau L (2017). Extremely efficient internal exciton dissociation through edge states in layered 2D perovskites. Science.

[33] Zhu H, Fu Y, Meng F, Wu X, Gong Z (2015). Lead halide perovskite nanowire lasers with low lasing thresholds and high-quality factors. Nat. Mater..

[34] Eaton SW, Lai M, Gibson NA, Wong AB, Dou L (2016). Lasing in robust cesium lead halide perovskite nanowires. Proc. Natl. Acad. Sci. USA.

[35] Wang N, Cheng L, Ge R, Zhang S, Miao Y (2016). Perovskite light-emitting diodes based on solution-processed self-organized multiple quantum wells. Nat. Photonics.

[36] Du KZ, Meng W, Wang X, Yan Y, Mitzi DB (2017). Bandgap engineering of lead-free double perovskite Cs_2_AgBiBr_6_ through trivalent metal alloying. Angew. Chem. Int. Ed..

[37] Saouma FO, Stoumpos CC, Wong J, Kanatzidis MG, Jang JI (2017). Selective enhancement of optical nonlinearity in two-dimensional organic-inorganic lead iodide perovskites. Nat. Commun..

[38] Yao EP, Yang Z, Meng L, Sun P, Dong S (2017). High-brightness blue and white LEDs based on inorganic perovskite nanocrystals and their composites. Adv. Mater..

[39] Abdi-Jalebi M, Andaji-Garmaroudi Z, Cacovich S, Stavrakas C, Philippe B (2018). Maximizing and stabilizing luminescence from halide perovskites with potassium passivation. Nature.

[40] Lin K, Xing J, Quan LN, de Arquer FPG, Gong X (2018). Perovskite light-emitting diodes with external quantum efficiency exceeding 20 percent. Nature.

[41] Tan Z, Wu Y, Hong H, Yin J, Zhang J (2016). Two-dimensional (C_4_H_9_NH_3_)_2_PbBr_4_ perovskite crystals for high-performance photodetector. J. Am. Chem. Soc..

[42] Wei H, Fang Y, Mulligan P, Chuirazzi W, Fang H-H (2016). Sensitive X-ray detectors made of methylammonium lead tribromide perovskite single crystals. Nat. Photonics.

[43] Deng W, Huang L, Xu X, Zhang X, Jin X (2017). Ultrahigh-responsivity photodetectors from perovskite nanowire arrays for sequentially tunable spectral measurement. Nano Lett..

[44] Liu Y, Zhang Y, Yang Z, Ye H, Feng J (2018). Multi-inch single-crystalline perovskite membrane for high-detectivity flexible photosensors. Nat. Commun..

[45] Adinolfi V, Ouellette O, Saidaminov MI, Walters G, Abdelhady AL (2016). Fast and sensitive solution-processed visible-blind perovskite uv photodetectors. Adv. Mater..

[46] Ma J, Fang C, Chen C, Jin L, Wang J (2019). Chiral 2D perovskites with a high degree of circularly polarized photoluminescence. ACS Nano.

[47] Zhao Y, Qiu Y, Feng J, Zhao J, Chen G (2021). Chiral 2D-perovskite nanowires for stokes photodetectors. J. Am. Chem. Soc..

[48] Fu Y, Yuan M, Zhao YJ, Dong MQ, Guo YW (2023). Gradient bandgap-tunable perovskite microwire arrays toward flexible color-cognitive devices. Adv. Funct. Mater..

[49] Zhang J, Wang Q, Zhang X, Jiang J, Gao Z (2017). High-performance transparent ultraviolet photodetectors based on inorganic perovskite CsPbCl_3_ nanocrystals. RSC Adv..

[50] Zhuang R, Wang X, Ma W, Wu Y, Chen X (2019). Highly sensitive X-ray detector made of layered perovskite-like (NH_4_)_3_Bi_2_I_9_ single crystal with anisotropic response. Nat. Photonics.

[51] Kim H, Kim RM, Namgung SD, Cho NH, Son JB (2022). Ultrasensitive near-infrared circularly polarized light detection using 3D perovskite embedded with chiral plasmonic nanoparticles. Adv. Sci..

[52] Huang X, Xiao X, Dong G (2020). Metal halide perovskites functionalized by patterning technologies. Adv. Mater. Technol..

[53] Zhan Y, Cheng Q, Song Y, Li M (2022). Micro-nano structure functionalized perovskite optoelectronics: from structure functionalities to device applications. Adv. Funct. Mater..

[54] Fang H, Li J, Ding J, Sun Y, Li Q (2017). An origami perovskite photodetector with spatial recognition ability. ACS Appl. Mater. Interfaces.

[55] Feng J, Yan X, Liu Y, Gao H, Wu Y (2017). Crystallographically aligned perovskite structures for high-performance polarization-sensitive photodetectors. Adv. Mater..

[56] Ji C, Dey D, Peng Y, Liu X, Li L (2020). Ferroelectricity-driven self-powered ultraviolet photodetection with strong polarization sensitivity in a two-dimensional halide hybrid perovskite. Angew. Chem. Int. Ed..

[57] Li CY, Chen C, Liu Y, Su J, Qi DX (2022). Multiple-polarization-sensitive photodetector based on a perovskite metasurface. Opt. Lett..

[58] Zhao Y, Li X, Feng J, Zhao J, Guo Y (2022). Chiral 1D perovskite microwire arrays for circularly polarized light detection. Giant.

[59] Sheng X, Chen G, Wang C, Wang W, Hui J (2018). Polarized optoelectronics of CsPbX_3_ (x = Cl, Br, I) perovskite nanoplates with tunable size and thickness. Adv. Funct. Mater..

[60] Zeng LH, Chen QM, Zhang ZX, Wu D, Yuan H (2019). Multilayered PdSe_2_/perovskite schottky junction for fast, self-powered, polarization-sensitive, broadband photodetectors, and image sensor application. Adv. Sci..

[61] Zhan Y, Wang Y, Cheng Q, Li C, Li K (2019). A butterfly-inspired hierarchical light-trapping structure towards a high-performance polarization-sensitive perovskite photodetector. Angew. Chem. Int. Ed..

[62] Ma J, Fang C, Liang L, Wang H, Li D (2021). Full-stokes polarimeter based on chiral perovskites with chirality and large optical anisotropy. Small.

[63] Li SX, Xia H, Wang L, Sun XC, An Y (2022). Self-powered and flexible photodetector with high polarization sensitivity based on MAPbBr_3_–MAPbI_3_ microwire lateral heterojunction. Adv. Funct. Mater..

[64] Zhang X, Yao Y, Liang L, Niu X, Wu J (2022). Self-assembly of 2D hybrid double perovskites on 3D Cs_2_AgBiBr_6_ crystals towards ultrasensitive detection of weak polarized light. Angew. Chem. Int. Ed..

[65] Xu X, Han Z, Zou Y, Li J, Gu Y (2021). Miniaturized multispectral detector derived from gradient response units on single MAPbX_3_ microwire. Adv. Mater..

[66] Leung SF, Ho KT, Kung PK, Hsiao VKS, Alshareef HN (2018). A self-powered and flexible organometallic halide perovskite photodetector with very high detectivity. Adv. Mater..

[67] Feng X, He Y, Qu W, Song J, Pan W (2022). Spray-coated perovskite hemispherical photodetector featuring narrow-band and wide-angle imaging. Nat. Commun..

[68] Cao F, Tian W, Wang M, Li L (2018). Polarized ferroelectric field-enhanced self-powered perovskite photodetector. ACS Photonics.

[69] Han S, Ma Y, Hua L, Tang L, Wang B (2022). Soft multiaxial molecular ferroelectric thin films with self-powered broadband photodetection. J. Am. Chem. Soc..

[70] Akkerman QA, Raino G, Kovalenko MV, Manna L (2018). Genesis, Challenges and opportunities for colloidal lead halide perovskite nanocrystals. Nat. Mater..

[71] Zhao Y, Qiu Y, Gao H, Feng J, Chen G (2020). Layered-perovskite nanowires with long-range orientational order for ultrasensitive photodetectors. Adv. Mater..

[72] Sun ME, Geng T, Yong X, Lu S, Ai L (2021). Pressure-triggered blue emission of zero-dimensional organic bismuth bromide perovskite. Adv. Sci..

[73] Oku T (2020). Crystal structures of perovskite halide compounds used for solar cells. Rev. Adv. Mater. Sci..

[74] Wang A, Yan X, Zhang M, Sun S, Yang M, Shen W (2016). Controlled synthesis of lead-free and stable perovskite derivative Cs_2_SnI_6_ nanocrystals via a facile hot-injection process. Chem. Mater..

[75] Zhou N, Bekenstein Y, Eisler CN, Zhang D, Schwartzberg AM (2019). Perovskite nanowire–block copolymer composites with digitally programmable polarization anisotropy. Sci. Adv..

[76] Kim YH, Zhai Y, Gaulding EA, Habisreutinger SN, Moot T (2020). Strategies to achieve high circularly polarized luminescence from colloidal organic-inorganic hybrid perovskite nanocrystals. ACS Nano.

[77] Lai M, Shin D, Jibril L, Mirkin CA (2022). Combinatorial synthesis and screening of mixed halide perovskite megalibraries. J. Am. Chem. Soc..

[78] Long G, Adamo G, Tian J, Klein M, Krishnamoorthy HNS (2022). Perovskite metasurfaces with large superstructural chirality. Nat. Commun..

[79] Steele JA, Pan W, Martin C, Keshavarz M, Debroye E (2018). Photophysical pathways in highly sensitive Cs_2_AgBiBr_6_ double-perovskite single-crystal X-ray detectors. Adv. Mater..

[80] Lu X, Li J, Zhang Y, Han Z, He Z (2022). Recent progress on perovskite photodetectors for narrowband detection. Adv. Photonics Res..

[81] Li L, Liu X, Li Y, Xu Z, Wu Z (2019). Two-dimensional hybrid perovskite-type ferroelectric for highly polarization-sensitive shortwave photodetection. J. Am. Chem. Soc..

[82] Yuan H, Liu X, Afshinmanesh F, Li W, Xu G (2015). Polarization-sensitive broadband photodetector using a black phosphorus vertical p–n junction. Nat. Nanotechnol..

[83] Wang J, Jiang C, Li W, Xiao X (2022). Anisotropic low-dimensional materials for polarization-sensitive photodetectors: From materials to devices. Adv. Opt. Mater..

[84] Li M, Han S, Teng B, Li Y, Liu Y (2020). Minute-scale rapid crystallization of a highly dichroic 2D hybrid perovskite crystal toward efficient polarization-sensitive photodetector. Adv. Opt. Mater..

[85] Han Z, Fu W, Zou Y, Gu Y, Liu J (2021). Oriented perovskite growth regulation enables sensitive broadband detection and imaging of polarized photons covering 300–1050 nm. Adv. Mater..

[86] Gao L, Zeng K, Guo J, Ge C, Du J (2016). Passivated single-crystalline CH_3_NH_3_PbI_3_ nanowire photodetector with high detectivity and polarization sensitivity. Nano Lett..

[87] Zhao Z, Li Y, Du Y, Zhang L, Wei J (2020). Preparation and testing of anisotropic MAPbI_3_ perovskite photoelectric sensors. ACS Appl. Mater. Interfaces.

[88] Li SX, Xia H, Liu TY, Zhu H, Feng JC (2023). In situ encapsulated moire perovskite for stable photodetectors with ultrahigh polarization sensitivity. Adv. Mater..

[89] Zhou F, Abdelwahab I, Leng K, Loh KP, Ji W (2019). 2D perovskites with giant excitonic optical nonlinearities for high-performance sub-bandgap photodetection. Adv. Mater..

[90] Dou L, Lai M, Kley CS, Yang Y, Bischak CG (2017). Spatially resolved multicolor CsPbX_3_ nanowire heterojunctions via anion exchange. Proc. Natl. Acad. Sci. USA.

[91] Wang Y, Fullon R, Acerce M, Petoukhoff CE, Yang J (2017). Solution-processed MoS_2_/organolead trihalide perovskite photodetectors. Adv. Mater..

[92] Fu Q, Wang X, Liu F, Dong Y, Liu Z (2019). Ultrathin ruddlesden–popper perovskite heterojunction for sensitive photodetection. Small.

[93] Chen Y, Lei Y, Li Y, Yu Y, Cai J (2020). Strain engineering and epitaxial stabilization of halide perovskites. Nature.

[94] Pan D, Fu Y, Spitha N, Zhao Y, Roy CR (2021). Deterministic fabrication of arbitrary vertical heterostructures of two-dimensional ruddlesden-popper halide perovskites. Nat. Nanotechnol..

[95] Chen J, Fu Y, Samad L, Dang L, Zhao Y (2017). Vapor-phase epitaxial growth of aligned nanowire networks of cesium lead halide perovskites (CsPbX_3_, X = Cl, Br, I). Nano Lett..

[96] Gao Y, Zhao L, Shang Q, Zhong Y, Liu Z (2018). Ultrathin CsPbX_3_ nanowire arrays with strong emission anisotropy. Adv. Mater..

[97] He X, Gao W, Xie L, Li B, Zhang Q (2016). Wafer-scale monodomain films of spontaneously aligned single-walled carbon nanotubes. Nat. Nanotechnol..

[98] Zhou Y, Luo J, Zhao Y, Ge C, Wang C (2018). Flexible linearly polarized photodetectors based on all-inorganic perovskite CsPbI_3_ nanowires. Adv. Opt. Mater..

[99] Saidaminov MI, Adinolfi V, Comin R, Abdelhady AL, Peng W (2015). Planar-integrated single-crystalline perovskite photodetectors. Nat. Commun..

[100] Lei Y, Chen Y, Zhang R, Li Y, Yan Q (2020). A fabrication process for flexible single-crystal perovskite devices. Nature.

[101] Pi Y, Zhao J, Zhao Y, Feng J, Zhang C (2020). Capillary-bridge controlled patterning of stable double-perovskite microwire arrays for non-toxic photodetectors. Front. Chem..

[102] Yuan M, Zhao Y, Feng J, Gao H, Zhao J (2022). Ultrasensitive photodetectors based on strongly interacted layered-perovskite nanowires. ACS Appl. Mater. Interfaces.

[103] Li SX, Xu YS, Li CL, Guo Q, Wang G (2020). Perovskite single-crystal microwire-array photodetectors with performance stability beyond 1 year. Adv. Mater..

[104] Zheng H, Ju B, Wang X, Wang W, Li M (2018). Circularly polarized luminescent carbon dot nanomaterials of helical superstructures for circularly polarized light detection. Adv. Opt. Mater..

[105] Kim NY, Kyhm J, Han H, Kim SJ, Ahn J (2019). Chiroptical-conjugated polymer/chiral small molecule hybrid thin films for circularly polarized light-detecting heterojunction devices. Adv. Funct. Mater..

[106] Wang Z, Gao M, Hao X, Qin W (2020). Helical-chiroptical nanowires generated orbital angular momentum for the detection of circularly polarized light. Appl. Phys. Lett..

[107] Zhu D, Jiang W, Ma Z, Feng J, Zhan X (2022). Organic donor-acceptor heterojunctions for high performance circularly polarized light detection. Nat. Commun..

[108] Dang Y, Liu X, Cao B, Tao X (2021). Chiral halide perovskite crystals for optoelectronic applications. Matter.

[109] Zhao Y, Dong M, Feng J, Zhao J, Guo Y (2021). Lead-free chiral 2D double perovskite microwire arrays for circularly polarized light detection. Adv. Opt. Mater..

[110] Wang L, Xue Y, Cui M, Huang Y, Xu H (2020). A chiral reduced-dimension perovskite for an efficient flexible circularly polarized light photodetector. Angew. Chem. Int. Ed..

[111] Zhang X, Liu X, Li L, Ji C, Yao Y (2021). Great amplification of circular polarization sensitivity via heterostructure engineering of a chiral two-dimensional hybrid perovskite crystal with a three-dimensional MAPbI_3_ crystal. ACS Cent. Sci..

[112] Zhang X, Weng W, Li L, Wu H, Yao Y (2021). Heterogeneous integration of chiral lead-chloride perovskite crystals with Si wafer for boosted circularly polarized light detection in solar-blind ultraviolet region. Small.

[113] Yang C-K, Chen W-N, Ding Y-T, Wang J, Rao Y (2019). The first 2D homochiral lead iodide perovskite ferroelectrics: [R- and S-1-(4-chlorophenylethylammonium]_2_PbI_4_. Adv. Mater..

[114] Dang Y, Liu X, Sun Y, Song J, Hu W (2020). Bulk chiral halide perovskite single crystals for active circular dichroism and circularly polarized luminescence. J. Phys. Chem. Lett..

[115] Jana MK, Song R, Liu H, Khanal DR, Janke SM (2020). Organic-to-inorganic structural chirality transfer in a 2D hybrid perovskite and impact on rashba-dresselhaus spin-orbit coupling. Nat. Commun..

[116] Zhao J, Zhao Y, Guo Y, Zhan X, Feng J (2021). Layered metal-halide perovskite single-crystalline microwire arrays for anisotropic nonlinear optics. Adv. Funct. Mater..

[117] Yu Z, Cao S, Zhao Y, Guo Y, Dong M (2022). Chiral lead-free double perovskite single-crystalline microwire arrays for anisotropic second-harmonic generation. ACS Appl. Mater. Interfaces.

[118] Zhao Y, Zhao J, Guo Y, Zhao J, Feng J (2022). Reversible phase transition for switchable second harmonic generation in 2D perovskite microwires. SmartMat.

[119] Long G, Jiang C, Sabatini R, Yang Z, Wei M (2018). Spin control in reduced-dimensional chiral perovskites. Nat. Photonics.

[120] Wang J, Fang C, Ma J, Wang S, Jin L (2019). Aqueous synthesis of low-dimensional lead halide perovskites for room-temperature circularly polarized light emission and detection. ACS Nano.

[121] Ishii A, Miyasaka T (2020). Direct detection of circular polarized light in helical 1D perovskite-based photodiode. Sci. Adv..

[122] Duim H, Loi MA (2021). Chiral hybrid organic-inorganic metal halides: a route toward direct detection and emission of polarized light. Matter.

[123] Ahn J, Lee E, Tan J, Yang W, Kim B (2017). A new class of chiral semiconductors: chiral-organic-molecule-incorporating organic-inorganic hybrid perovskites. Mater. Horiz..

[124] Li D, Liu X, Wu W, Peng Y, Zhao S (2021). Chiral lead-free hybrid perovskites for self-powered circularly polarized light detection. Angew. Chem. Int. Ed..

[125] Zhang X, Ye H, Liang L, Niu X, Wu J (2022). Direct detection of near-infrared circularly polarized light via precisely designed chiral perovskite heterostructures. ACS Appl. Mater. Interfaces.

[126] Li W, Coppens ZJ, Besteiro LV, Wang W, Govorov AO (2015). Circularly polarized light detection with hot electrons in chiral plasmonic metamaterials. Nat. Commun..

[127] Gholipour B, Adamo G, Cortecchia D, Krishnamoorthy HN, Birowosuto MD (2017). Organometallic perovskite metasurfaces. Adv. Mater..

[128] Cui T, Bai B, Sun HB (2019). Tunable metasurfaces based on active materials. Adv. Funct. Mater..

[129] Huang C, Zhang C, Xiao S, Wang Y, Fan Y (2020). Ultrafast control of vortex microlasers. Science.

[130] Joo W-J, Kyoung J, Esfandyarpour M, Lee S-H, Koo H (2020). Metasurface-driven oled displays beyond 10,000 pixels per inch. Science.

[131] Klein M, Wang Y, Tian J, Ha ST, Paniagua-Dominguez R (2022). Polarization-tunable perovskite light-emitting metatransistor. Adv. Mater..

[132] Dai M, Wang C, Qiang B, Wang F, Ye M (2022). On-chip mid-infrared photothermoelectric detectors for full-stokes detection. Nat. Commun..

[133] Hu X, Zhang X, Liang L, Bao J, Li S (2014). High-performance flexible broadband photodetector based on organolead halide perovskite. Adv. Funct. Mater..

[134] Alshamrani N, Grieco A, Hong B, Fainman Y (2021). Miniaturized integrated spectrometer using a silicon ring-grating design. Opt. Express.

[135] Lin Q, Armin A, Burn PL, Meredith P (2015). Filterless narrowband visible photodetectors. Nat. Photonics.

[136] Shen L, Fang Y, Wei H, Yuan Y, Huang J (2016). A highly sensitive narrowband nanocomposite photodetector with gain. Adv. Mater..

[137] Li L, Deng Y, Bao C, Fang Y, Wei H (2017). Self-filtered narrowband perovskite photodetectors with ultrafast and tuned spectral response. Adv. Opt. Mater..

[138] Fang Y, Dong Q, Shao Y, Yuan Y, Huang J (2015). Highly narrowband perovskite single-crystal photodetectors enabled by surface-charge recombination. Nat. Photonics.

[139] Higashi Y, Kim K-S, Jeon H-G, Ichikawa M (2010). Enhancing spectral contrast in organic red-light photodetectors based on a light-absorbing and exciton-blocking layered system. J. Appl. Phys..

[140] Cicek E, McClintock R, Cho CY, Rahnema B, Razeghi M (2013). Al_x_Ga_1-x_N-based back-illuminated solar-blind photodetectors with external quantum efficiency of 89%. Appl. Phys. Lett..

[141] Sobhani A, Knight MW, Wang Y, Zheng B, King NS (2013). Narrowband photodetection in the near-infrared with a plasmon-induced hot electron device. Nat. Commun..

[142] Li J, Wang J, Ma J, Shen H, Li L (2019). Self-trapped state enabled filterless narrowband photodetections in 2D layered perovskite single crystals. Nat. Commun..

[143] Zhang MN, Wu X, Riaud A, Wang XL, Xie F (2020). Spectrum projection with a bandgap-gradient perovskite cell for colour perception. Light Sci. Appl..

[144] Sun H, Tian W, Wang X, Deng K, Xiong J (2020). In situ formed gradient bandgap-tunable perovskite for ultrahigh-speed color/spectrum-sensitive photodetectors via electron-donor control. Adv. Mater..

[145] Wang XL, Chen Y, Chu Y, Liu WJ, Zhang DW (2022). Spectrum reconstruction with filter-free photodetectors based on graded-band-gap perovskite quantum dot heterojunctions. ACS Appl. Mater. Interfaces.

[146] Pan Q, Su M, Zhang Z, Chen B, Huang Z (2020). Omnidirectional photodetectors based on spatial resonance asymmetric facade via a 3D self-standing strategy. Adv. Mater..

[147] Dong Y, Zou Y, Song J, Zhu Z, Li J (2016). Self-powered fiber-shaped wearable omnidirectional photodetectors. Nano Energy.

[148] Shen L, Fang Y, Wang D, Bai Y, Deng Y (2016). A self-powered, sub-nanosecond-response solution-processed hybrid perovskite photodetector for time-resolved photoluminescence-lifetime detection. Adv. Mater..

[149] Lu H, Tian W, Cao F, Ma Y, Gu B (2016). A self-powered and stable all-perovskite photodetector-solar cell nanosystem. Adv. Funct. Mater..

[150] Su L, Zhao Z, Li H, Yuan J, Wang Z (2015). High-performance organolead halide perovskite-based self-powered triboelectric photodetector. ACS Nano.

[151] Han S, Li M, Liu Y, Guo W, Hong MC (2021). Tailoring of a visible-light-absorbing biaxial ferroelectric towards broadband self-driven photodetection. Nat. Commun..

[152] Shaikh PA, Shi D, Retamal JRD, Sheikh AD, Haque MA (2016). Schottky junctions on perovskite single crystals: light-modulated dielectric constant and self-biased photodetection. J. Mater. Chem. C.

[153] Sun H, Tian W, Cao F, Xiong J, Li L (2018). Ultrahigh-performance self-powered flexible double-twisted fibrous broadband perovskite photodetector. Adv. Mater..

[154] Fang H, Li Q, Ding J, Li N, Tian H (2016). A self-powered organolead halide perovskite single crystal photodetector driven by a dvd-based triboelectric nanogenerator. J. Mater. Chem. C.

[155] Cen G, Liu Y, Zhao C, Wang G, Fu Y (2019). Atomic-layer deposition-assisted double-side interfacial engineering for high-performance flexible and stable CsPbBr(3) perovskite photodetectors toward visible light communication applications. Small.

[156] Shen K, Xu H, Li X, Guo J, Sathasivam S (2020). Flexible and self-powered photodetector arrays based on all-inorganic CsPbBr(3) quantum dots. Adv. Mater..

[157] Saidaminov MI, Haque MA, Almutlaq J, Sarmah S, Miao X-H (2017). Inorganic lead halide perovskite single crystals: phase-selective low-temperature growth, carrier transport properties, and self-powered photodetection. Adv. Opt. Mater..

